# A conserved ATP- and Scc2/4-dependent activity for cohesin in tethering DNA molecules

**DOI:** 10.1126/sciadv.aay6804

**Published:** 2019-11-27

**Authors:** Pilar Gutierrez-Escribano, Matthew D. Newton, Aida Llauró, Jonas Huber, Loredana Tanasie, Joseph Davy, Isabel Aly, Ricardo Aramayo, Alex Montoya, Holger Kramer, Johannes Stigler, David S. Rueda, Luis Aragon

**Affiliations:** 1 Cell Cycle Group, Medical Research Council London Institute of Medical Sciences, Du Cane Road, London W12 0NN, UK.; 2 Single Molecule Imaging Group, Medical Research Council London Institute of Medical Sciences, Du Cane Road, London W12 0NN, UK.; 3 Molecular Virology, Department of Medicine, Imperial College London, Du Cane Road, London W12 0NN, UK.; 4 LUMICKS, De Boelelaan 1085, 1081 HV, Amsterdam, Netherlands.; 5 Gene Center, Ludwig-Maximilians-University, 81377 Munich, Germany.; 6 Microscopy Facility, Medical Research Council London Institute of Medical Sciences, Du Cane Road, London W12 0NN, UK.; 7 Biological Mass Spectrometry and Proteomics Facility, Medical Research Council London Institute of Medical Sciences, Du Cane Road, London W12 0NN, UK.

## Abstract

Sister chromatid cohesion requires cohesin to act as a protein linker to hold chromatids together. How cohesin tethers chromatids remains poorly understood. We have used optical tweezers to visualize cohesin as it holds DNA molecules. We show that cohesin complexes tether DNAs in the presence of Scc2/Scc4 and ATP demonstrating a conserved activity from yeast to humans. Cohesin forms two classes of tethers: a “permanent bridge” resisting forces over 80 pN and a force-sensitive “reversible bridge.” The establishment of bridges requires physical proximity of dsDNA segments and occurs in a single step. “Permanent” cohesin bridges slide when they occur in trans, but cannot be removed when in cis. Therefore, DNAs occupy separate physical compartments in cohesin molecules. We finally demonstrate that cohesin tetramers can compact linear DNA molecules stretched by very low force (below 1 pN), consistent with the possibility that, like condensin, cohesin is also capable of loop extrusion.

## Teaser

Reconstitution of ATP-dependent cohesion by purified yeast and human cohesin complexes shows distinct types of cohesin bridges.

## INTRODUCTION

The establishment of sister chromatid cohesion is essential for accurate chromosome segregation during the mitotic cell cycle. Cohesin is a complex of the SMC (structural maintenance of chromosomes) family originally identified for its role in tethering sister chromatids from S phase until anaphase (*1*, *2*). In addition to its function in sister chromatid cohesion, cohesin modulates the organization of interphase nuclei and mitotic chromosomes (*1*, *3*, *4*). Studies in vertebrates have shown that cohesin complexes maintain contacts between different loci in cis and in this way contribute to the folding of individual chromatids into distinct loops that provide an integral level of genome architecture (*1*, *3*, *4*). The current model for how SMC complexes, including cohesin, might form DNA loops involves the capture and bending of DNA segments followed by progressive enlargement of these to form loops (*5*, *6*); this activity has been termed “loop extrusion.” Evidence for this model has been obtained from in vitro analysis of purified yeast condensin (*7*). Cohesin’s most prominent function is the tethering of sister chromatids, which is expected to involve an ability to bridge two DNA molecules in trans. Unlike condensin, cohesin has not yet been demonstrated to extrude loops in vitro. A potential activity in loop extrusion has been suggested for cohesin because of its involvement in the maintenance of cis looping and as a potential linear tracking mechanism that could explain the preferential use of convergent CTCF DNA motifs at TAD borders during genome folding (*8*, *9*). However, it is currently not clear how loop extrusion could explain the well-established role of cohesin in sister chromatid cohesion.

Mechanistically, we only have a vague idea of how cohesin might generate intermolecular tethers while mediating sister chromatid cohesion. Two main models have been proposed to explain cohesin function in sister chromatid cohesion: the “ring” or “embrace” model (*10*, *11*), in which a single cohesin ring entraps both sister DNA molecules (*10*), and the “handcuff model,” where sister chromatid cohesion is mediated by the entrapment of sister DNAs in different cohesin complexes and a subsequent cohesin-cohesin interaction (*1*, *12*, *13*). The capture of the pair of double-stranded DNA (dsDNA) molecules during the establishment of sister chromatid cohesion by a single cohesin molecule in the “embrace model” has been proposed to occur by either (i) passage of the replisomes through the ring lumen of a DNA-bound cohesin or (ii) when a DNA-bound cohesin captures a single-stranded DNA (ssDNA) at the fork, which is then converted into dsDNA by DNA synthesis (*14*). Although cohesin complexes have been purified from fission yeast (*15*), frogs (*16*), and human cells (*17*), single-molecule analyses of DNA bridging activities have not been reported. Purified cohesin complexes have been shown to exhibit DNA binding activity in a salt-resistant manner (*18*) and to rapidly diffuse on DNA; however, these were shown to be independent of adenosine triphosphate (ATP) (*15*–*17*), suggesting that they are not at the core of its ATP-dependent activity.

Single-molecule studies of purified yeast condensin have shown that this SMC complex compacts DNA molecules on magnetic tweezers (*19*), translocates along linear DNA molecules in an ATP-dependent manner (*20*), and forms DNA loop–like structures on surface-tethered, flow-stretched DNA (*7*). Furthermore, while purified condensin exhibits robust ATPase activity in the presence of DNA (*19*), purified yeast cohesin is a poor ATPase on its own (*21*, *22*). Recent work has shown that the Scc2-Scc4 loader complex greatly stimulates cohesin’s ATPase activity (*21*, *22*). On the basis of these findings, we sought to investigate activities of budding yeast cohesin in the presence of the Scc2-Scc4 loader complex using the following two complementary single-molecule approaches: DNA curtains and optical tweezers.

## RESULTS

### Purification of cohesin complexes

**Fig. 1:**
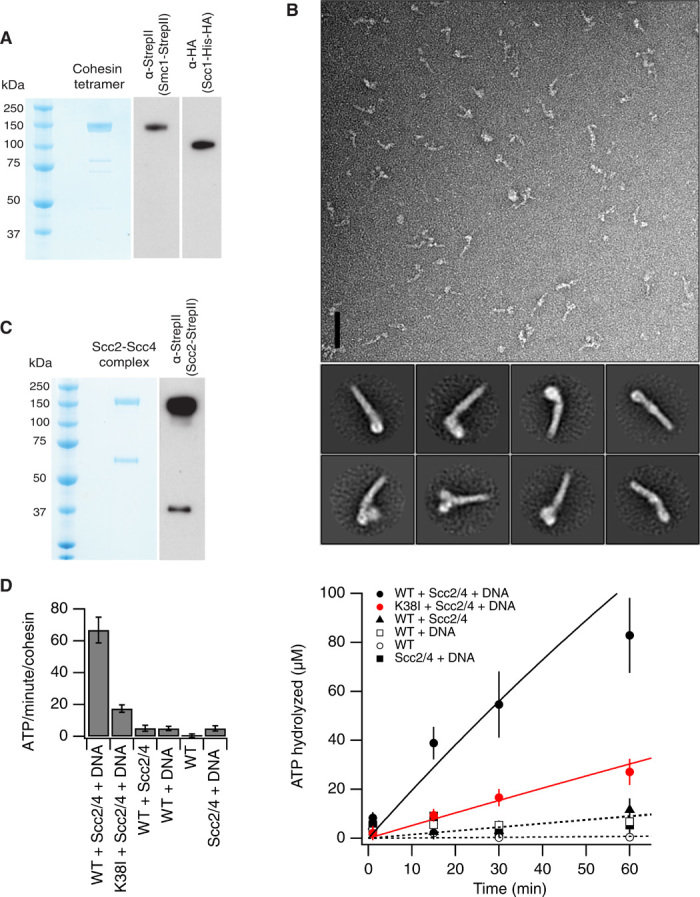
Biochemical purification of yeast cohesin. (**A**) Purified cohesin tetramer containing Smc1, Smc3, Scc1, and Scc3 was analyzed by SDS–polyacrylamide gel electrophoresis (PAGE) followed by Coomassie blue staining. Western blot analysis shows the mobility of Smc1 and Scc1. (**B**) Top panel: Representative micrograph of a BS3-crosslinked cohesin sample observed in negative stain EM. Scale bar, 50 nm. Bottom panel: Class averages obtained with RELION. A set of the best ~5000 particles was used for this classification. The size of the circular mask is 450 Å. (**C**) Coomassie blue staining of the purified Scc2-Scc4 complex. (**D**) ATP hydrolysis by yeast cohesin and cohesin ATPase mutant Smc3-K38I with or without the Scc2-Scc4 complex.

To investigate activities of yeast cohesin using single-molecule assays, we first purified budding yeast cohesin tetramers, containing Smc1, Smc3, Scc1/Mcd1 (thereafter referred to as Scc1), and Scc3, from exponentially growing yeast cultures (Fig. 1A). Cohesin subunits were overexpressed in high-copy plasmids using galactose (*GAL*)–inducible promoters. Purified material was obtained via affinity chromatography, using a triple-StrepII tag fused to the Smc1 subunit, followed by passage through a HiTrap Heparin HP column (Fig. 1A and table S1). Analysis of purified complexes by negative-stain electron microscopy confirmed the presence of rod-shaped cohesin holocomplexes, the majority in a folded conformation (Fig. 1B) (*23*). The Scc2-Scc4 complex was also purified from budding yeast (Fig. 1C) using a similar strategy and showed DNA binding activity as expected (fig. S1A) (*21*, *22*). Purified cohesin also bound plasmid DNA in a salt-resistant manner (fig. S1B), and the bound plasmid was released by DNA cleavage with restriction enzymes (fig. S1C). This is consistent with the topological binding mode proposed for this complex (*18*, *22*). However, in our hands, this activity was not strictly dependent on ATP and was not stimulated by Scc2-Scc4 (fig. S1B), in contrast to what has been reported recently (*18*, *22*). Last, we confirmed that our purified Scc2-Scc4 complex was able to stimulate cohesin ATPase activity (Fig. 1D) (*21*, *22*).

### Activity of yeast cohesin tetramers on double-tethered DNA curtains

**Fig. 2:**
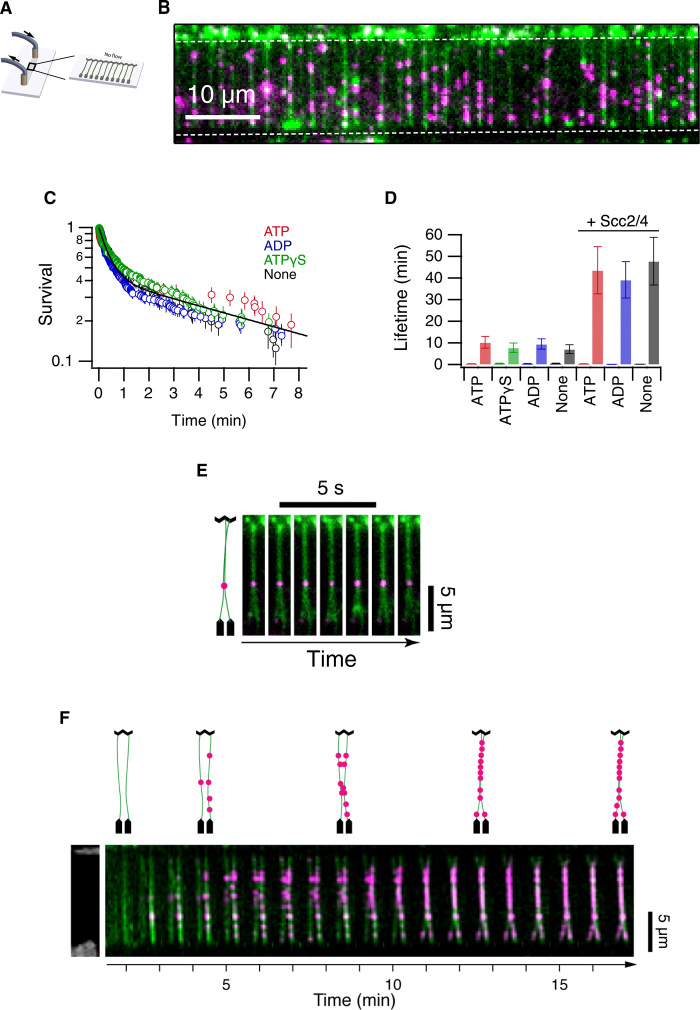
Analysis of yeast cohesin on DNA curtains. (**A**) Schematic representation of double-tethered DNA curtains used in the study. (**B**) Image of cohesin tagged with quantum dots (magenta) bound to λ-DNA stained with YOYO-1 (green). Scale bar, 10 μm. (**C**) Survival probability plots of cohesin in the presence of ATP, ADP, ATPγS, or no nucleotide. (**D**) Lifetimes of cohesin (fast phase and slow phase) in the presence or absence of Scc2-Scc4 and different ATP analogs. Error bars are 68% confidence intervals from bootstrapping. (**E**) Image of a pair of double-tethered DNA curtains bound by cohesin. DNA molecules are in green, and cohesin is in magenta. Diagrammatic representation is shown (left). (**F**) Time-lapse images of a pair of double-tethered DNA curtains bound by cohesin as they are tethered. DNA molecules are in green, and cohesin is in magenta. Diagrammatic representation is shown (top). Pairing events were observed frequently in the DNA curtains. An average of 5 to 10 events per DNA curtain was detected.

Next, we sought to test whether budding yeast cohesin exhibited the behavior described for cohesin from other organisms on DNA curtains (*15*–*17*). λ-DNA molecules (48.5 kb) were anchored to a lipid bilayer in a flow cell surface and aligned into double-tethered DNA curtains using nanofabricated barriers (Fig. 2A) (*15*). Quantum dots (Qdots) conjugated to antibodies against the hemagglutinin tag (HA3) fused to the C-terminal region of the Scc1 kleisin subunit were used to visualize the complexes (Fig. 2B). On flowing the labeled cohesin complex over the DNA curtains, binding was observed at low ionic strength (Fig. 2A). The chamber was flushed with a high ionic strength buffer to remove nontopologically bound complexes (Fig. 2A). While a large fraction of cohesin complexes dissociated, we observed diffusion along the DNA (Fig. 2B). The binding preference of cohesin to more A/T-rich regions reported earlier (*15*) was also observed (Fig. 2, C to E). The diffusion coefficients correlated with the ionic strength of the buffer (fig. S2F). The survival probabilities of cohesin were not affected by the addition of ATP, or the ATP analogs adenosine 5′-diphosphate (ADP) and ATPγS (Fig. 2C). We found that the presence of Scc2-Scc4 enhanced the ability of cohesin to stay bound on the DNA (Fig. 2D); however, the presence of nucleotides did not alter cohesin stabilities (Fig. 2D). Therefore, these results are consistent with the activities observed for cohesin from other organisms (*15*, *17*) and show that budding yeast cohesin undergoes rapid diffusion on DNA curtains in an ATP-independent manner.

In our DNA curtain experiments, we made an observation not reported in earlier studies (*15*, *17*). Cohesin signals were often observed bound between what appeared to be two fused DNAs (Fig. 2E). The pairing events formed under low-salt conditions in the presence of ATP (Fig. 2F and movies S1 and S2), but they persisted when the chamber was flushed with a high ionic strength buffer, raising the possibility that topologically bound complexes mediated these events.

### Cohesin can form two classes of DNA bridges in an ATP- and Scc2-Scc4–dependent manner

To further explore our observation that cohesin tetramers paired λ-DNA molecules on the DNA curtains, we decided to use a dual-trap optical tweezer with confocal fluorescence microscopy capabilities. A similar approach has been previously used in the study of protein-DNA interactions (*24*). Briefly, we tether a λ-DNA molecule with biotinylated ends to two optically trapped streptavidin-coated polystyrene beads, enabling us to accurately apply and measure forces on the captured DNA molecule. We performed our experiments in multichannel laminar flow cells where we had the possibility to move the tethered DNA to different flow lanes containing different protein complexes and buffers. In addition, we were able to image the tethered DNAs using confocal fluorescence microscopy. Overall, the approach allows increased experimental control over DNA curtains. Proteins can be added, removed, or incubated under different salt conditions sequentially, and the physical effect of their activities can be measured accurately on a single DNA molecule.

**Fig. 3:**
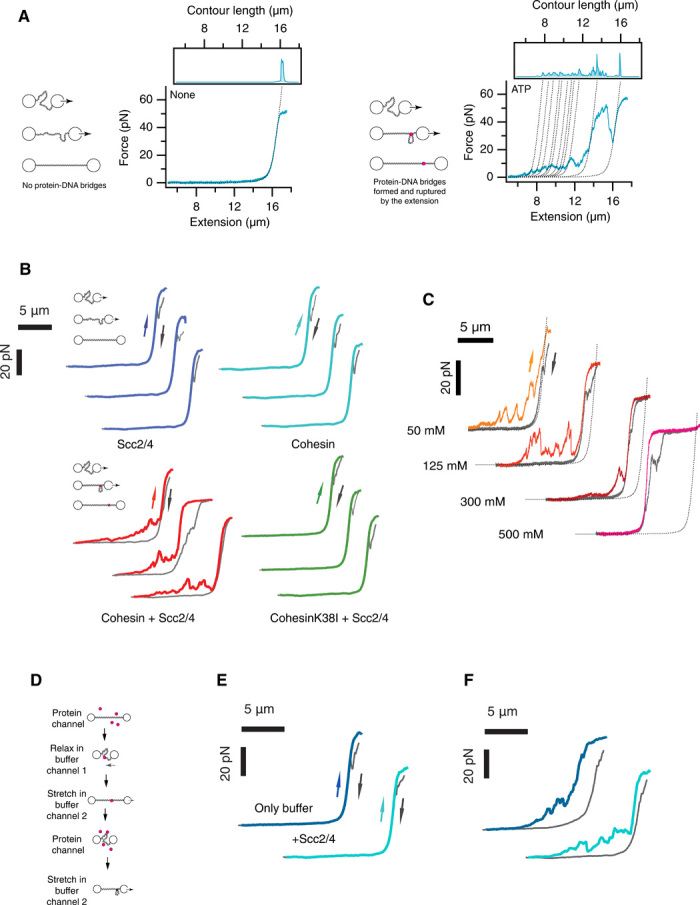
Cohesin bridges DNA in an ATP- and Scc2-Scc4–dependent manner. (**A**) Schematic representation of FE curve for λ-DNA exhibiting the presence (right diagram and graph) and absence (left diagram and graph) of protein DNA bridges. Dotted line is fit to worm-like chain for naked DNA. (**B**) FE curves for λ-DNAs preincubated with 1 nM cohesin and 2.5 nM Scc2-Scc4 complex and 1 mM ATP (Cohesin + Scc2/4), 1 nM cohesin and 1 mM ATP (Cohesin), 2.5 nM Scc2-Scc4 complex and 1 mM ATP (Scc2/4), or 1 nM cohesin ATPase mutant and 2.5 nM Scc2-Scc4 complex and 1 mM ATP (CohesinK38I + Scc2/4). Schematic diagram of the experimental design. After capturing a single DNA molecule between two optically trapped beads, DNA was incubated in the presence of protein in a relaxed conformation (3-μm bead distance) for 30 s in 50 mM NaCl and then moved to a buffer channel with 125 mM NaCl for extension and measurements. Only incubation with 1 nM cohesin and 2.5 nM Scc2-Scc4 complex and 1 mM ATP (Cohesin + Scc2/4) showed DNA bridging rupture events. (**C**) FE curves in the presence of increasing ionic strength. High salt favors topologically constrained and permanent DNA bridges. (**D**) Schematic representation of the experimental design to test cohesin second DNA capture. After capture of λ-DNA between the two optically trapped beads, DNA is extended and incubated for 30 s in the protein channel. DNA is moved to a buffer channel and then relaxed (3-μm bead distance) and incubated for 30 s before reextension to test for DNA bridges (E). The extended DNA is then incubated in a relaxed position in the protein channel and then moved to buffer channel and extended to confirm that bridges can be formed when protein is loaded while DNA is relaxed (F). (**E**) λ-DNA incubated with 1 nM cohesin, 2.5 nM Scc2-Scc4 complex, and 1 mM ATP in an extended conformation and then moved to a buffer channel (125 mM NaCl) in the presence of 1 mM ATP (buffer only, dark blue) or 2.5 nM Scc2-Scc4 complex and 1 mM ATP (+Scc2/4, light blue). DNAs were reextended, and the FE curves were recorded. (**F**) The λ-DNA molecules in (E) were incubated in a relaxed position (3-μm bead distance) in the presence of 1 nM cohesin, 2.5 nM Scc2-Scc4 complex, and 1 mM ATP DNAs. DNAs were moved to a buffer-only channel (125 mM NaCl containing 1 mM ATP) and reextended. FE curves show the presence of DNA bridging rupture events.

To test for the formation of intramolecular cohesin bridges in cis, we adapted a previously published protocol that measures protein-mediated DNA bridging (Fig. 3A) (*25*, *26*). First, we captured a single λ-DNA molecule and generated a force-extension (FE) curve in the absence of protein by extending the molecule slightly beyond its contour length (~16 μm). We then moved the DNA to a channel containing 1 nM cohesin, 2.5 nM Scc2-Scc4 complex, and 1 mM ATP in 50 mM NaCl and incubated for 30 s in a relaxed conformation (~3 μm between beads). Following incubation, the relaxed DNA was then moved to a channel without protein but containing 1 mM ATP in 125 mM NaCl. Reextending the DNA in the buffer-only channel yielded FE curves with sawtooth features at extensions shorter than the contour length (Fig. 3B, Cohesin + Scc2/4). This is characteristic of intramolecular bridge rupture events (*25*, *26*) (Fig. 3A, right) and shows that cohesin can tether the DNA in cis forming a protein-mediated bridge between different segments of the molecule, thus creating an intramolecular loop. When we repeated this protocol in the presence of 1 nM cohesin and no Scc2-Scc4 (Fig. 3B, Cohesin), or 2.5 nM Scc2-Scc4 and no cohesin (Fig. 3B, Scc2/4), FE curves identical to those of the initial naked DNA were observed, demonstrating that no protein-mediated bridges were formed (Fig. 3A, left). Similarly, incubating 1 nM cohesin and 2.5 nM Scc2-Scc4 complex in the absence of ATP, or with the ATP analogs ADP or ATPγS, yielded FE curves identical to those of naked DNA (fig. S3A). To confirm the requirement of ATP, we repeated the protocol in the presence of 1 nM cohesin ATPase mutant (K38I) (fig. S4) and 2.5 nM Scc2-Scc4 (Fig. 3B, CohesinK38I + Scc2/4). FE curves identical to those of the naked DNA were observed (Fig. 3B, CohesinK38I + Scc2/4). Therefore, the DNA bridging activity requires ATP and depends on the Scc2-Scc4 loader complex.

Next, we tested the effect of ionic strength on cohesin bridging (Fig. 3C). Cohesin bridges were observed at all salt concentrations tested (Fig. 3C). The length of DNA extension released during the rupture of a DNA bridge can be directly related to the loop size encompassed by the bridge. We analyzed the sizes of the DNA bridges from the FE curves at 125 mM salt (fig. S3B) and found that the distribution of loop sizes is exponential with a characteristic size of ~900 base pairs (bp), consistent with a model of random bridge formation (*5*, *6*). Most of the small sawtooth peaks observed at low forces and extensions disappear under high-salt conditions, while the overall contour length of the DNA remained reduced (Fig. 3C). We also recorded FE curves when we relaxed tethers (Fig. 3, B and C, reverse arrows) after the extensions are done in the buffer channel, therefore in the absence of protein (Fig. 3, B and C, forward arrows). These showed that compaction due to DNA bridges formed at low-salt concentrations was lost after extension (Fig. 3C, reverse arrows; 50 mM NaCl) with force. However, relaxation of tethers with DNA bridges formed at high-salt concentrations showed compaction events that had resisted after extension (Fig. 3C, reverse arrows; 300 and 500 mM NaCl). These results show two distinct types of cohesin bridging events: (i) one predominantly occurring at low salt that is characterized by frequent interactions that are “reversible” and can be disrupted by moderate force (5 to 40 pN) and (ii) a second “permanent” bridge class that resists higher ionic strength conditions and full physical stretching of the DNA molecule. Both classes of DNA bridges were not observed when an ATPase mutant cohesin complex (SMC3-K38I) was used (Fig. 3B, CohesinK38I + Scc2/4), confirming that the ATPase activity of the complex is a requirement for both types of bridges. Next, we tested whether permanent bridges could resist repeated extensions. We performed two cycles of bead extension and relaxation and confirmed the persistence of the permanent cohesin bridge using FE curves (fig. S5). We conclude that permanent cohesin bridges resist high stretching forces and that the complexes mediating the tethers cannot be displaced from the DNA molecules. This explains the repeated detection of the same bridge on FE curves during the cycle of bead extension and relaxation (fig. S5).

### The tethering of two dsDNA segments by cohesin occurs in a single-capture step

Recent studies using purified cohesin from *Schizosaccharomyces pombe* have shown that cohesin can capture a second DNA, but only if single stranded (*14*). This led the authors to conclude that cohesin is not capable of trapping to dsDNAs in vitro (*14*). Moreover, it was suggested that this activity is likely to occur at replication forks, where cohesin bound to a dsDNA molecule is exposed to nascent ssDNA (*14*). The second capture of the single-stranded molecule was dependent on the presence of cohesin loader and ATP (*14*). Our results seem to contradict this because we show that cohesin purified from *Saccharomyces cerevisiae* is fully able to trap two dsDNA molecules (Fig. 3, B and C). Next, we decided to investigate whether capture of the two molecules is sequential or simultaneous. In our original tethering assay, we could not differentiate whether the two dsDNAs are captured sequentially or in a single step, as we had incubated the DNA in a relaxed position (with the two DNA segments in proximity). To distinguish whether one or two events were involved in the formation of the cohesin tethers observed, we sought to test whether cohesin could capture a second DNA after initial loading. To this aim, we captured a single λ-DNA molecule and generated an FE curve. We maintained the DNA in an extended position (~15 μm between beads) using a pulling force of 5 pN (Fig. 3D) and loaded cohesin by moving the DNA to a channel containing 1 nM cohesin, 2.5 nM Scc2-Scc4 complex, and 1 mM ATP in 50 mM NaCl. We incubated the DNA for 30 s (Fig. 3D) before moving it to a different channel containing 1 mM ATP in 125 mM NaCl. We then relaxed the DNA conformation (~3 μm between beads) to allow DNA segments to come into proximity (Fig. 3D) and incubated in the relaxed conformation for an additional 30 s. The FE curve obtained after reextension of the DNA was identical to the initial naked DNA profile (Fig. 3E, Only buffer, and fig. S6). We obtained a similar result when we included 2.5 nM Scc2-Scc4 complex and 1 mM ATP in the channel where we relaxed the DNA (Fig. 3E, +Scc2/4, and fig. S6). These results show that loaded cohesin is unable to capture a second DNA segment. To confirm that DNA bridges could be formed in the same DNA in one step, we relaxed the molecules used in the experiments and incubated them for 30 s in a channel containing 1 nM cohesin, 2.5 nM Scc2-Scc4 complex, and 1 mM ATP. When molecules were reextended, the resulting FE curves confirmed the formation of DNA bridges (Fig. 3F and fig. S6). In addition, we confirmed that cohesin complexes can bind to extended DNAs using a published DNA friction protocol (fig. S7) (*27*). Therefore, our results are consistent with a previous report (*14*), showing that cohesin bound to DNA cannot undergo a second capture event involving a dsDNA molecule, but demonstrate that cohesin is able to capture two dsDNAs simultaneously. A previous study could not evaluate the possibility that cohesin could capture two dsDNAs simultaneously, thus reaching an erroneous conclusion (*14*). We conclude that cohesin establishes bridges between two dsDNA in a single step, or two kinetically very close steps, which requires physical proximity of the DNA segments.

### Cohesin can slide while tethering two DNAs intermolecularly

**Fig. 4:**
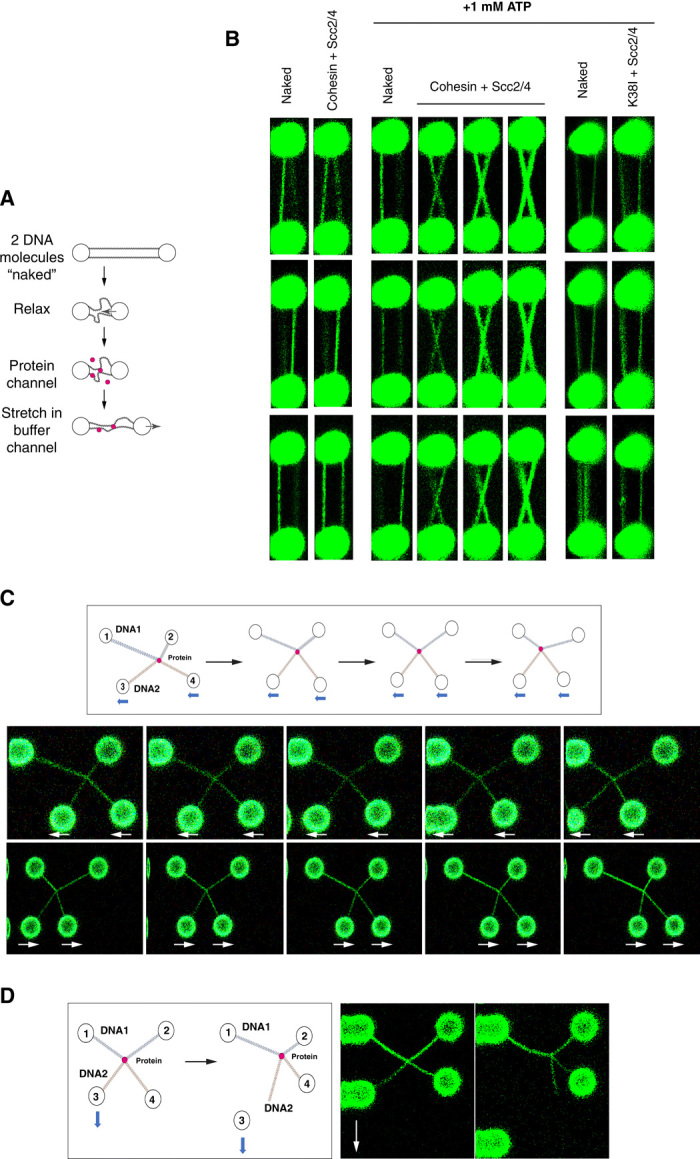
Cohesin and Scc2-Scc4 mediate intermolecular DNA bridges that slide on DNAs. (**A**) Schematic representation of the experimental design for the dual-trap optical tweezer to generate permanent intermolecular cohesin bridges. Two λ-DNA molecules are tethered between the two beads and incubated in a relaxed position (3-μm bead distance) in the presence or absence of protein in buffer containing 50 mM NaCl. The relaxed molecules are then moved to a different channel containing 300 mM NaCl and reextended. Imaging is done before incubations and after reextension in a buffer containing 300 mM NaCl and 50 nM SYTOX Orange to visualize DNA. (**B**) Two λ-DNA molecules were tethered and treated as described in (A) and incubated with either (i) 1 nM cohesin, 2.5 nM Scc2-Scc4, and no ATP (Cohesin + Scc2/4, left); (ii) 1 nM cohesin, 2.5 nM Scc2-Scc4, and 1 mM ATP (Cohesin + Scc2/4, middle); or (iii) 1 nM cohesin ATPase mutant K38I, 2.5 nM Scc2-Scc4, and 1 mM ATP (K38I + Scc2/4, right). Imaging was performed before incubation and after DNA reextension in a buffer containing 300 mM NaCl to minimize DNA entanglement and 50 nM SYTOX Orange to visualize DNA. Images from three independent experiments are shown. Three independent experiments are shown for each category. (**C**) Schematic representation of the experimental design to test for sliding of permanent cohesin bridges (top diagram). Following the formation of an intermolecular cohesin bridge (see fig. S8 for details in bridge formation protocol), beads 3 and 4 were moved together in the *x* axis to slide the bridge along DNA1. Images showing two representative sliding experiments are shown. Experiments were performed in a buffer containing 300 mM NaCl and 50 nM SYTOX Orange. Movies of the experiments are shown in movies S4 and S5. The experiment was performed three times, and sliding was observed in all cases. (**D**) Schematic representation of the experimental design to disrupt intermolecular cohesin bridges. Following the formation of an intermolecular cohesin bridge, bead 3 is moved down in the *y* axis until one of the DNA ends loses contact with the bead. Imaging was performed before and after the pull in a buffer containing 300 mM NaCl and 50 nM SYTOX Orange. Representative experiment is shown. A movie of the experiment is shown in movie S6.

Next, we investigated whether cohesin can form intermolecular bridges. We developed an intermolecular bridging assay, where two dsDNA molecules are tethered in parallel between the pair of beads, and tested the ability of cohesin to form bridges between these two molecules (Fig. 4A). DNA molecules were visualized with SYTOX Orange. After confirming the presence of two DNA molecules tethered in parallel between the beads (Fig. 4B, Naked), the DNA was incubated in a relaxed state to bring the DNAs into proximity (~3-μm bead distance) in the presence of 1 nM cohesin and 2.5 nM Scc2-Scc4 and 1 mM ATP for 30 s. The DNAs were moved to a buffer-only channel (300 mM NaCl plus 1 mM ATP). Strikingly, clear bridging was observed between the two molecules on reextension (Fig. 4B, Cohesin + Scc2/4). DNA bridges did not form in the absence of ATP (Fig. 3B, no ATP) or when we used cohesin ATPase mutant complex (Fig. 4B, K38I + Scc2/4), confirming that cohesin’s ATPase activity is required. Bridge formation in this assay was very efficient; of 10 molecules tested, 8 showed intermolecular bridges (Fig. 4B, Cohesin + Scc2/4) and 2 showed intramolecular bridging on the two individual DNAs. Intermolecular bridges always appeared to be near the midpoint of the DNA (Fig. 4B, Cohesin + Scc2/4). Potential reasons to explain this include the fact that the central region of λ-DNA molecules is rich in A/T content where cohesin might bind preferentially. Alternatively, cohesin might be able to slide on the DNA while maintaining tethers and therefore likely to move to the center regions as the molecules are extended. To further characterize this, we used a quadruple-trap optical tweezer setup, which allows the independent manipulation of the two DNA molecules (*27*).

We first captured two single λ-DNA molecules using a pair of traps for each (DNA1 between traps 1 and 2 and DNA2 between traps 3 and 4) in a parallel conformation (fig. S8). Both DNA molecules were stretched close to their contour lengths (~16 μm). We then manipulated DNA2 using beads 3 and 4 and moved it upward (in the *z* direction) before rotating it 90° and moving it into a crossed conformation directly above DNA1 (fig. S8). We then lowered DNA2 to its original *z* position and relaxed it to ensure physical contact between the two DNA molecules at the junction point (fig. S8). We then moved the crossed DNAs into a different channel containing 1 nM cohesin, 2.5 nM Scc2-Scc4, and 1 mM ATP (60 s, 50 mM NaCl) before returning the DNAs to a channel containing 1 mM ATP in 300 mM NaCl. We reversed the manipulation of DNA2, first moving bead 3 upward and over DNA1 before manipulating beads 3 and 4 so that DNA2 was rotated −90° and lowered back to the original position where DNA1 and DNA2 were parallel to each other. When we moved the beads to a channel containing SYTOX Orange to visualize DNA, we observed that DNA1 and DNA2 were bridged (fig. S8), as expected from our analysis of parallel DNA bridging in the dual-trap optical tweezers setup (Fig. 4B, Cohesin + Scc2/4 + 1 mM ATP). We then tested whether simultaneously moving DNA2 using beads 3 and 4 in the *x* axis would cause the sliding of the bridge along DNA1 (Fig. 4C). We observed that the bridge could be moved, showing that cohesin can slide on DNAs while tethering two DNA molecules in trans (Fig. 4C and movies S4 and S5). When we applied force to disrupt the bridge [moving bead 3 down in the *y* axis (away from beads 1 and 2); Fig. 4D], we were not able to break apart the cohesin tether. At high forces, the interaction between the ends of the DNAs and the beads often snapped (Fig. 4D and movie S6). Amazingly, cohesin bridges resisted this, and half of DNA2 could be observed hanging from the bridge (Fig. 4D and movie S6). We conclude that permanent intermolecular cohesin bridges can slide on DNA and resist high force. We predict that the forces exerted to disrupt the interaction between the DNAs and the bead exceed 80 pN. At these high forces, the prediction is that all the protein interfaces on cohesin rings should be disrupted. Therefore, cohesin association with DNA in permanent tethers is likely to occur in a manner that resists opening of the interfaces.

### Cohesin DNA-tethering activity is conserved in human cohesin tetramers containing Stag1

**Fig. 5:**
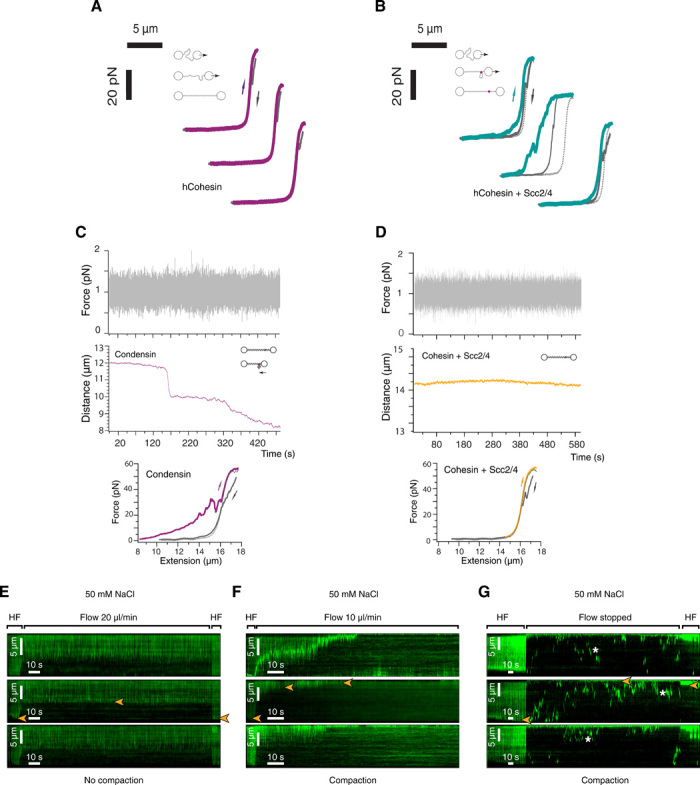
Cohesin does not compact linear DNA molecules stretched under low force. (**A**) FE curve for λ-DNA preincubated with 1 nM human cohesin and 1 mM ATP in 125 mM NaCl buffer (hCohesin). Dotted line is fit to worm-like chain model. After capturing a single DNA molecule between two optically trapped beads, DNA was incubated in the presence of protein in 50 mM NaCl buffer in a relaxed conformation (3-μm bead distance) for 30 s and then moved to the 125 mM NaCl buffer channel for extension and measurements. No evidence of DNA bridges was observed under this condition. (**B**) FE curve for λ-DNA preincubated with 1 nM human cohesin, 2.5 nM yeast Scc2-Scc4, and 1 mM ATP in 125 mM NaCl buffer (hCohesin + Scc2/4). Experimental procedure as in (A). FE curves exhibited multiple rupture events indicating the presence of reversible and permanent DNA bridges. (**C**) DNA compaction trace for λ-DNA molecule extended using a force of 1 pN (top). The DNA was tethered between two beads. One bead was clamped (fixed), while a 1-pN force was applied to the second bead to maintain the molecule extended. The DNA was then incubated in the presence of 1 nM condensin (1 mM ATP in 50 mM NaCl) (left, magenta trace). The FE curve for the λ-DNA full extension after incubation is shown (bottom). Additional examples can be found in fig. S10. (**D**) DNA compaction trace for λ-DNA molecule extended using a force of 1 pN (top) in the presence of 1 nM cohesin and 2.5 nM Scc2-Scc4 complex (1 mM ATP in 50 mM NaCl) (right, yellow trace). The distance between the beads was recorded over time. The FE curve for the λ-DNA full extension after incubation is shown (bottom). Additional examples can be found in fig. S10. (**E**) Kymograms of single-tethered λ-DNA stained with (YOYO-1) during the incubation with yeast cohesin and Scc2-Scc4 in the presence of ATP in 50 mM NaCl buffer at a flow rate of 20 μl/min. HF, high flow. The free end of DNA is marked with orange arrowheads. No compaction of single-tethered λ-DNAs was observed. (**F**) Kymograms of single-tethered λ-DNA stained with (YOYO-1) during the incubation with yeast cohesin and Scc2-Scc4 in the presence of ATP in 50 mM NaCl buffer at a flow rate of 10 μl/min. The conditions are as in (E) except for the reduced flow rate. Slow compaction of single-tethered λ-DNAs was observed over time (orange arrowheads mark the free end of DNA). (**G**) Kymograms of single-tethered λ-DNA stained with (YOYO-1) during the incubation with yeast cohesin and Scc2-Scc4 in the presence of ATP in 50 mM NaCl buffer at stopped flow. The free end of DNA is marked with orange arrowheads. The HF phase at the end of the experiment shows that the DNA was compacted during the stopped flow phase. Note that under stopped flow conditions, DNA molecules that diffuse laterally on the flow chip can transiently cross the field of view and also appear in a kymogram representation. Examples are marked with asterisks (*). These events bear no relevance for the interpretations of the assay.

Previous studies using purified cohesin from different organisms did not report DNA bridging activities (*15*–*17*); however, the studies did not use budding yeast cohesin. We therefore decided to test whether the bridging activity observed is specific for *S. cerevisiae* cohesin tetramers or it has been conserved in cohesin from other organisms. To this aim, we purified the human cohesin (hCohesin) tetramer complex, containing hSmc1, hSmc3, hRad21, and Stag1, as described previously (fig. S9A) (*28*). We then tested whether hCohesin could bridge DNA intramolecularly. We captured a single λ-DNA molecule and generated an FE curve in the absence of protein to confirm the presence of naked DNA. We then moved the DNA to a channel containing 1 nM hCohesin and 1 mM ATP in 50 mM NaCl and incubated it for 30 s in a relaxed conformation (~3 μm between beads). We then moved the relaxed DNA to a channel without protein in the presence of 1 mM ATP in 125 mM NaCl. Reextending the DNA resulted in FE curves with a naked DNA profile (Fig. 5A, hCohesin), demonstrating that hCohesin on its own cannot promote DNA bridges. Although we could not obtain hScc2-Scc4, we decided to test whether the budding yeast loader complex Scc2-Scc4 (scScc2-Scc4) had any effect on hCohesin activity. To this aim, we repeated the intramolecular DNA bridging assays with hCohesin and included the Scc2-Scc4 loader complex in the incubations. Relaxed DNA was incubated in the presence of 1 nM hCohesin tetramer, 2.5 nM scScc2-Scc4 complex, and 1 mM ATP in 50 mM NaCl. The relaxed DNA was then moved to a channel with 1 mM ATP in 125 mM NaCl. Reextension yielded the sawtooth features characteristic of intramolecular bridge rupture events (Fig. 5B, hCohesin + Scc2/4) detected with yeast cohesin tetramers (Fig. 3C, 125 mM). Therefore, hCohesin tetramers containing Stag1 have conserved the ability to bridge DNA. hCohesin was able to form both reversible and permanent bridges (Fig. 4B, hCohesin + Scc2/4).

### Cohesin compacts linear DNA molecules stretched by very low forces

Besides mediating sister chromatid cohesion (*1*, *2*), cohesin holds individual chromatids in cis, thus forming loops (*4*, *29*, *30*). Recently, yeast condensin was the first SMC complex shown to exhibit an activity compatible with loop extrusion (*7*). It is unclear whether this activity is also present in the other eukaryotic SMC complexes cohesin and Smc5/6. Condensin can compact linear DNA against forces of up to 2 pN (*19*). However, condensin loop extrusion activity is only observed when DNA is stretched under significantly lower forces (below 0.5 pN) (*7*). We purified yeast condensin (fig. S9, B and C) using an established protocol (*20*, *31*) and tested whether, as predicted from studies using magnetic tweezers (*19*), it could also compact λ-DNA molecules extended in the optical tweezers against a force of 1 pN. A single λ-DNA molecule was first captured between the beads. We then immobilized one of the beads and applied a constant force of 1 pN to the other bead in the opposite direction. This maintains the DNA extended with ~14 μm between beads. We then moved the DNA to a channel containing 1 nM condensin in 50 mM NaCl buffer supplemented with 1 mM ATP. We incubated the extended DNA recording the distance between the two beads over time (Fig. 5C, Condensin). We observed progressive decrease of the distance between the beads (Fig. 5C, Condensin, and fig. S10), consistent with the activity of condensin as a motor that compacts DNA (*19*). Some condensation events occurred in short bursts and caused the molecule to shorten ~1 to 2 μm in a few seconds (Fig. 5C, Condensin, and fig. S10). After incubation, we generated an FE curve, which showed the presence of sawtooth peaks characteristic of protein-mediated DNA bridging (Fig. 5C, bottom) (*25*, *26*). Condensin bridges were fully reversible and disappeared when the DNA was extended (Fig. 5C, bottom). It is unclear whether the compaction observed is due to loop extrusion because this activity was reported to occur at forces below 1 pN (*7*). Next, we sought to text whether yeast cohesin tetramers could also compact extended λ-DNA molecules in this assay. We incubated the DNA extended using 1 pN of force with 1 nM cohesin, 2.5 nM Scc2-Scc4 complex, and 1 mM ATP in 50 mM NaCl buffer and incubated the extended DNA recording the distance between the two beads (Fig. 5D, Cohesin). The distance between the beads did not change over time (Fig. 5D, Cohesin, and fig. S10); therefore, we conclude that cohesin tetramers do not exhibit DNA compaction activity in this assay. As expected, the FE curve generated after incubation showed no evidence of protein-mediated DNA bridging (Fig. 5D, bottom), and similar results were obtained when we used a stretching force of 0.5 pN.

Since loop extrusion activity of condensin occurs at forces below 0.5 pN (*7*), we considered the possibility that yeast cohesin might also be able to extrude loops (and hence condense DNA) at extremely low forces. Below 0.5 pN, our optical tweezer did not reliably maintain the distance between the beads (data not shown). We therefore used single-tethered DNA curtains and different flow rates to extend DNA at very low tensions. Initially, we incubated cohesin in the presence of Scc2/Scc4 and ATP using a 125 mM NaCl buffer and a flow rate of 30 μl/min; however, we did not observe compaction of single-tethered DNAs (data not shown). We then decided to reduce the ionic strength of the buffer to 50 mM NaCl and the flow rate to 20 μl/min (Fig. 5E). We did not observe compaction in the course of the experiment (Fig. 5E). However, when we further reduced the flow rate to 10 μl/min we observed slow compaction of the majority of the molecules (Fig. 5F). Last, we performed the same experiment but stopped the flow after protein injection (Fig. 5G). We observed rapid compaction of the single-tethered DNAs (Fig. 5G). From these data, we conclude that DNA compaction in single-tethered DNA curtains at such low flow is likely to be formed as a consequence of compaction that might involve loop extrusion since this activity only occurs at low ionic strength conditions and when DNA is extended by very low force.

## DISCUSSION

The original role attributed to cohesin was the maintenance of sister chromatid cohesion from S phase until the anaphase onset (*1*, *2*). Here, we have developed powerful single-molecule assays to probe the mechanisms by which cohesin holds DNAs together. Using them, we have shown that cohesin complexes can form different types of bridges between dsDNAs and that this requires Scc2-Scc4 and ATP. The two classes of cohesin tethers exhibited different physical properties, particularly the sensitivity to being broken by force. The reversible bridges were disrupted when moderate forces (5 to 40 pN) were applied (Fig. 3C). In contrast, permanent bridges could withstand extreme forces without being disrupted (Figs. 3C and 4D). They are also more predominant in high ionic strength conditions (Fig. 3C). On the basis of these physical properties, we propose that permanent bridges represent cohesin complexes that maintain sister chromatid cohesion. However, further characterization of their genesis, architecture, and biochemistry will be important to confirm such proposal. Reversible bridges were more predominant at low-salt concentrations (Fig. 3C), which suggest that they are likely formed by protein-protein interactions. In low salt, cohesin is likely to be saturated on DNA and being relatively sticky could easily engage in nonspecific interactions. Therefore, some reversible bridging events could potentially represent nonspecific protein aggregation. In particular, this might be the case for intramolecular DNA bridging at 50 mM NaCl salt (Fig. 3C). However, even under these conditions, reversible bridges were ATP and Scc2/4 dependent (fig. S3A). At 125 mM NaCl salt, which is in the physiological range, reversible bridges were also significant and resisted forces of up to 40 pN (Fig. 3C), strongly arguing that reversible bridges are biologically relevant. Previous studies have demonstrated that cohesin can use nontopological mechanisms (*32*); in addition, interallelic complementation between different cohesin alleles has also been reported (*33*). It is therefore possible that reversible DNA bridges reflect functional cohesin-cohesin interactions.

Recent studies have interrogated cohesin mechanisms using biochemical reconstitution of topological loading onto plasmids (*14*, *18*, *34*, *35*). We believe that the single-molecule assay presented in this study is more informative for the study of cohesin bridging. In our hands, cohesin loading in the gel-based assay was not strictly ATP dependent and was not stimulated by Scc2/4, as observed for *S. pombe* cohesin (*14*, *18*, *34*, *35*). Topological loading efficiency can be dependent on multiple factors, but critically on the amount of protein used, the times of incubation, and the number and stringency of the washes. We followed the original protocol described for *S. pombe* cohesin (*18*), and despite attempting different conditions, we never observed ATP-dependent loading. It is therefore likely that *S. pombe* and *S. cerevisiae* cohesins behave differently. The observation that *S. pombe* cohesin does not show bridging activity in double-tethered DNA curtains (*15*), while *S. cerevisiae* cohesin does (Fig. 2, E and F), supports this possibility.

**Fig. 6:**
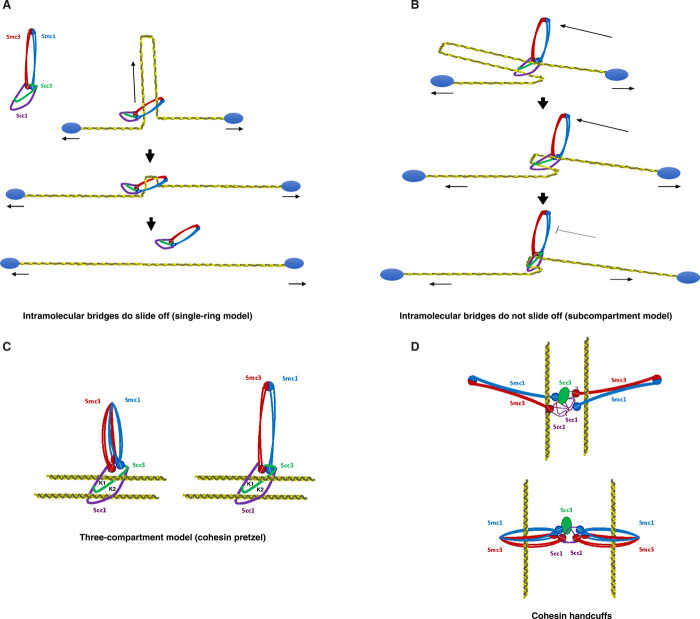
Tentative models for permanent cohesin bridges. (**A**) Schematic representation of expected behavior of intramolecular cohesin tethers from the previously proposed ring model. The model proposes that cohesin co-entraps two DNAs within its ring structure, i.e., both DNAs occupy one physical space within cohesin. From this model, it is expected that cohesin should be fully displaced from λ-DNA molecules when tethering in cis as force is applied to separate the beads. This is not what it was observed experimentally (Fig. 3C and fig. S5). (**B**) Schematic representation of expected behavior of intramolecular cohesin tethers from the subcompartment model. The subcompartment model is based on the assumption that DNAs are located in different physical compartments. The prediction from the model is that cohesin cannot be fully displaced from λ-DNA molecules when tethering them in cis. This is what we observed experimentally (Fig. 3C and fig. S5). (**C**) Proposed model for a single cohesin complex with at least three subcompartments (cohesin pretzel). In this model, sister DNAs occupy two different chambers (K1 and K2) of the K (kleisin) compartment formed between the SMC ATPase heads and the Scc1 subunit (*36*). Two possible conformations of SMC hinges are shown. Note that the experimental data are also compatible with the possibility that both DNAs jointly travel through the two chambers (K1 and K2) of the K compartment. (**D**) Schematic representation of previously proposed cohesin handcuffs models holding sister DNAs in different compartments of two separate complexes, which also fits with our experimental observations.

Our results using two DNA molecules demonstrate that permanent cohesin tethers can slide when force is applied (Fig. 4C); however, when the permanent bridges occur in cis, cohesin complexes cannot slide off the DNA molecules (Fig. 3C and fig. S5). The simplest explanation is that the two DNA molecules tethered are not located in the same physical space within the protein (Fig. 6A). The two main models proposed to explain how cohesin holds sister chromatids are the “ring” and “handcuff” models. The basic difference between these two models is the fact that in the ring model, the two DNAs occupy the same physical space within cohesin, i.e., they are co-entrapped in one compartment of the cohesin structure (*10*, *11*), while in the handcuff model (and all its variations), the two DNAs are located in different physical compartments (*1*, *12*, *13*), generally argued to be two separate (but interacting) complexes. On the basis of the single-ring model, it would be expected that cohesin slides off DNA molecules when bridging them intramolecularly (Fig. 6A). In contrast, our observations suggest that this is not the case (Fig. 3C). Using in vivo cysteine cross-linking of trimer cohesin complexes, it has been recently shown that cohesin has different subcompartments (*36*). Sister DNAs occupied the K (kleisin) compartment formed between the SMC ATPase heads and the Scc1 subunit (*36*). Scc3 and Scc1 form a module that binds DNA and is necessary for cohesin association to chromosomes (*37*), but Scc3 was not crosslinked in the subcompartment study (*36*). We propose that DNAs in permanent cohesin bridges might be held in two chambers of the K (kleisin) compartment (Fig. 6B, K1 and K2), physically separated by Scc3 (Fig. 6B), and the architecture would resemble a “pretzel-like” structure (Fig. 6B). The DNAs might be separated (one in K1 and the other in K2), or might travel through the two K compartments together. Alternatively, different compartments of two cohesin complexes might be involved (Fig. 6C).

Kimura *et al.* (*5*) first proposed that the SMC complex condensin might generate DNA loops (*5*). This was conceived as one of two models that could explain how condensin specifically produced (+) trefoil knots in the presence of a type II topoisomerase (*5*). The proposal was based on an earlier model of “loop expansion” that was put forward for bacterial MutS action (*38*). MutS loop expansion was shown to occur as a consequence of ATP-dependent bidirectional movement of the MutS dimer from the initial loading site (*38*). The proposal of Kimura *et al.* (*5*) has been recently demonstrated directly through the observation of condensin-dependent DNA loop–like structures on surface-tethered, flow-stretched DNA (*7*). The loop extrusion activity of cohesin was also conceived as a model that could explain the role of cohesin in genome folding through cis looping and the preferential use of convergent CTCF DNA motifs at TAD borders (*8*, *9*). We detected DNA compaction by yeast cohesin tetramers at very low flow rates (Fig. 5, F and G), as would be predicted from a loop extrusion activity similar to the one shown for condensin (*7*). HiC data show that removal of cohesin leads to loss of contacts at TAD boundaries (*6*, *8*, *39*), demonstrating that the complex is involved in the formation or maintenance of loops. It is likely that cohesin extrudes DNA loops in a similar manner to condensin (*7*). However, our data, although consistent with cohesin function as a loop extruder, do not demonstrate it. We would like to note that our data showing intramolecular tethering by cohesin do not imply that cohesin generates loops in vivo through random DNA bridging. We feel that this would be highly unlikely. The intramolecular tethers observed might reflect an in vitro activity (as cohesin is unlikely to differentiate between cis and trans tethering when loaded onto DNA in these assays). Further experiments will be required to test whether intramolecular tethering is of any relevance in vivo. The activities described here are fully consistent with the original role attributed to cohesin in maintaining sister chromatid cohesion (*1*, *2*). Our work provides a new critical tool for future investigations to further decipher how cohesin executes one of the critical functions required for genome inheritance, i.e., maintaining sister chromatids in close proximity from the time they are born in S phase until they are separated in anaphase.

## MATERIALS AND METHODS

### Protein expression and purification

The different subunits of the *S. cerevisiae* Scc2-Scc4 and cohesin complexes were synthesized under the control of galactose-inducible promoters and cloned into multicopy episomal vectors (*URA3-SCC4-GAL1-10promoter-SCC2*-3xmyc-3xStrepII*;TRP1*-*SMC1*-3xStrepII-*GAL1-10promoter*-*SMC3 GAL7promoter-MCD1*-8xHis-3xHA; *URA3-GAL1-10promoter-SCC3*). Yeast W303-1a strains carrying the different constructs (CCG14800 for the Scc2-Scc4 complex, CCG14801 for cohesin tetramer, and CCG14815 for cohesin *smc3*-K38I tetramer) were grown at 30°C in selective dropout medium containing 2% raffinose and 0.1% glucose to an OD_600_ (optical density at 600 nm) of 1. Protein expression was induced by addition of 2% galactose, and cells were grown for further 16 hours at 20°C. Cells were then harvested by centrifugation at 4°C, resuspended in two volumes of buffer A [25 mM Hepes (pH 7.5), 200 mM NaCl, 5% glycerol, 5 mM β-mercaptoethanol] containing 1× cOmplete EDTA-free protease inhibitor mix (Roche), frozen in liquid nitrogen, and lysed in Freezer-Mill (SPEX CertiPrep 6870). Cell powder was thawed at 4°C for 2 hours before mixing it with one volume of buffer A containing benzonase (Millipore) and incubated at 4°C for an extra hour. Cell lysates were clarified by centrifugation at 45,000*g* for 1 hour followed by filtration using 0.22-μm syringe filters. Clarified lysates were loaded onto 5-ml StrepTrap-HP columns (GE Healthcare) preequilibrated with buffer A. The resin was washed with five column volumes of buffer A and eluted with buffer B (buffer A containing 5 mM desthiobiotin). The peak fractions containing the overexpressed proteins were pooled together, and salt concentration was adjusted to 150 mM NaCl using 100 mM NaCl buffer A. Samples were then filtered as described above to remove residual aggregates and loaded onto 5-ml HiTrap Heparin HP (GE Healthcare) columns preequilibrated with 150 mM NaCl buffer A. Elution was carried out using a linear gradient from 150 mM to 1 M NaCl in buffer A. Peak fractions were pooled and concentrated by centrifugal ultrafiltration (100 kDa Amicon Ultra, Millipore). Salt concentration was adjusted to 300 mM NaCl during the concentration step. Gel filtration was carried out using a Superose 6 Increase 100/300 GL column (GE Healthcare) in 300 mM NaCl buffer A. Fractions corresponding to monomeric complexes were pooled and concentrated as described above. Purified proteins were analyzed by SDS-PAGE (NuPAGE 4 to 12% bis-tris protein gels, Thermo Fisher Scientific) and Coomassie staining (InstantBlue, Expedeon). Protein identification was carried out by mass spectrometry analysis and Western blot. *S. cerevisiae* condensin complex was expressed and purified, as previously described (*20*, *31*).

Human cohesin tetramer was purified, as described before (*28*). Human cohesin subunits (RAD21, SMC1A, SMC3-FLAG, 10xHis-SA1) were coexpressed in High Five insect (BTI-Tn-5B1-4) cells. Cells were disrupted by short sonication. Afterwards, the lysate was clarified by high-speed centrifugation. The complex was then purified via HisTrap [washing buffer: 25 mM tris (pH 7.5), 500 mM NaCl, 5% glycerol, 2 mM MgCl_2_, 20 mM imidazole, 0.01% Tween-20, 20 mM β-mercaptoethanol; elution buffer: 25 mM tris (pH 7.5), 150 mM NaCl, 5% glycerol, 2 mM MgCl_2_, 150 mM imidazole, 0.01% Tween-20]. Fractions were pooled and dialyzed [25 mM tris (pH 7.5), 150 mM NaCl, 5% glycerol, 2 mM MgCl_2_]. The protein was further purified by tandem ion exchange chromatography by using an anion-exchange column connected to a cation exchange column. The complex was then eluted from the cation-exchange column [25 mM tris (pH 7.5), 1 M NaCl, 5% glycerol, 2 mM MgCl_2_]. Subsequently, the peak fractions were pooled and dialyzed into storage buffer [25 mM tris (pH 7.5), 150 mM NaCl, 5% glycerol, 2 mM MgCl_2_]. Purity was confirmed by gel electrophoresis and mass spectrometry.

### Electrophoretic gel mobility shift assay

Increasing concentrations of the Scc2-Scc4 complex ranging from 100 to 800 nM were incubated for 45 min with 50 ng of pUC19 at 30°C in 25 mM tris-HCl (pH 7.0), 50 mM NaCl, 8% glycerol, bovine serum albumin (BSA; 0.1 mg/ml), and 0.5 mM dithiothreitol (DTT) in a final volume of 15 μl. The reactions were resolved by electrophoresis for 1 hour at 80 V on 0.8% (w/v) tris-acetate-EDTA (TAE) agarose gels at 4°C. DNA was detected on a fluorescent image analyzer (FLA-5000, Fujifilm) after SYBR Green I (Invitrogen, Thermo Fisher Scientific) gel staining. Condensin assays were carried out as previously described (*20*).

### Protein cross-linking and electron microscopy

For cross-linking of cohesin complex, protein samples were incubated with BS3 at a 1:3000 molar ratio in buffer XL [25 mM Hepes, 125 mM NaCl, 5% glycerol, 1 mM DTT (pH 8)] for 2 hours on ice before quenching with 10 mM tris-HCl (pH 8) for 30 min on ice.

Negative-stain grids were prepared as follows: 3.5 μl of suspended sample (final concentration of 0.02 mg/ml in buffer XL) was deposited on glow-discharged grids coated with a continuous carbon film. The sample was left on the grid for 1 min before blotting the excess liquid. A 3.5-μl drop of 2% uranyl acetate solution was added for 1 min, the stain was blotted away, and the grids were left to dry.

A set of 250 micrographs was collected on a Philips CM200 TWIN FEG electron microscope operated at 160 kV. Images were recorded on a Tietz 2k charge-coupled device camera at a nominal magnification of 38,000 and a final pixel size of 3.58 Å, contrast transfer function (CTF) parameters were estimated using Gctf (*40*). A total of ~9000 particles were automatically picked using Gautomatch software using class averages obtained from a manually picked subset of 1500 particles as references. The following two-dimensional classifications were performed with RELION v3.0 beta (*41*).

### In vitro cohesin loading assay

Cohesin loading assays were done as described in (*14*) using the pUC19 plasmid. Topologically bound DNA-cohesin complexes were immunoprecipitated using a μMACS HA Isolation kit (Miltenyi Biotec). Following incubation with Pst I and/or protein digestion, the recovered DNA was analyzed by electrophoresis on a 0.8% (w/v) TAE agarose gel in 1× TAE and visualized as described above.

### ATPase assays

For the ATPase assays, 30 nM cohesin was incubated at 29°C with 60 nM Scc2/4 and 0.2 nM λ-DNA (New England Biolabs) in ATPase buffer [35 mM tris-HCl (pH 7.0), 20 mM NaCl, 0.5 mM MgCl_2_, 13.3% glycerol, 0.003% Tween-20, 1 mM tris(2-carboxyethyl)phosphine (TCEP), BSA (0.2 mg/ml)]. The reaction was started by adding 400 μM ATP spiked with [γ-^32^P]ATP. One microliter of samples was taken after 1, 15, 30, and 60 min. The reaction was immediately stopped by adding 1 μl of 50 mM EDTA before spotting the samples on polyethyleneimine cellulose F sheets. The free phosphate was separated from ATP using thin-layer chromatography with 0.5 M LiCl, 1 M formic acid as the mobile phase. The spots were detected on a phosphor imager and analyzed using ImageJ. Data points were corrected for spontaneous ATP hydrolysis. Each reaction was performed in triplicate. Data were fitted to Michaelis-Menten kinetics.

### Single-molecule experiments

DNA curtain experiments were performed as described previously (*42*). Briefly, flow cells were produced by deposition of chromium features onto fused silica microscope slides by e-beam lithography. Flow cells were connected to a microfluidics system based on a syringe pump (Landgraf GmbH) and two injection valves (Idex) and illuminated by 488- or 561-nm lasers (Coherent) in a prism-type total internal reflection fluorescence (TIRF) configuration on an inverted microscope (Nikon Ti2e). Imaging was performed using an electron multiplying charge-coupled device (EMCCD) camera (Andor iXon life) with illumination times of 100 ms. λ-DNA (NEB) was end-modified by hybridizing biotinylated or digoxigeninated oligos complementary to the cos site and purified by size exclusion chromatography. Modified λ-DNA was anchored to the surface of a lipid bilayer in flow cells by biotin-streptavidin-biotin interactions, stretched by flow across chromium barriers, and anchored to downstream chromium pedestals by the digoxigenin-binding protein DIG10.3 (*43*). Experiments were performed in buffer M [40 mM tris-HCl (pH 7.8), 1 mM MgCl_2_, 1 mM DTT, BSA (1 mg/ml), 0.16 nM YOYO-1]. Cohesin complexes were labeled by incubating them at a concentration of 3 nM in a small volume of buffer M supplemented with 50 mM NaCl with 3× molar excess Qdots (SiteClick 705 kit, Invitrogen) fused to anti-HA antibodies (3F10, Roche) for 30 min at 4°C. The mixture was then supplemented with 8 nM Scc2/Scc4, 100 μm biotin, and 0.5 mM nucleotide (ATP, ADP, or ATPγS), if required, before injection. For diffusion measurements, the flow cell was flushed after the completion of loading with buffer M supplemented with KCl at the indicated concentrations and the flow was stopped. Illuminations were performed either continuously (diffusion and lifetime measurements) or with lower frame rates (intermolecular bridging videos). To minimize photodamage, 488-nm pulses to illuminate the DNA, if required, were only used at every 10th illumination.

Videos were recorded in NIS Elements (Nikon) and analyzed using custom-written software in Igor Pro (WaveMetrics). Lifetime measurements and initial binding distributions of cohesin complexes on DNA were generated by manually analyzing kymograms. Survival curves were generated by a Kaplan-Meier estimator, bootstrapped, and fitted to a double-exponential model.

For the determination of diffusion coefficients, labeled cohesin complexes were tracked using custom-written software, and the diffusion coefficients were extracted using a maximum-likelihood estimator (*44*), as described previously (*15*).

Optical tweezers experiments were carried out on C-trap and Q-trap systems integrating optical tweezers, confocal fluorescence microscopy, and microfluidics and recorded using BlueLake software (LUMICKS). The laminar flow cell was passivated using 0.50% pluronic and BSA (2 mg/ml). Biotin-labeled double-stranded λ-DNA molecules were tethered between two streptavidin-coated polyesterene beads (4.42 μm in diameter, Spherotech). Depending on the experiment, one or two individual double-stranded λ-DNA molecules were attached between two beads. The beads were previously passivated with BSA (1 mg/ml). After DNA capture, beads were incubated inside the protein channel either in a relaxed (~3 μm apart) or extended position (force clamp at 5 pN, ~14 μm apart) for 30 s and then returned to the buffer channel for FE, force clamp, and fluorescence analysis. Cohesin and Scc2-Scc4 complex were used at 1 nM and 2.5 nM concentrations, respectively. Beads and DNA catching and protein loading were performed in a buffer containing 50 mM tris-HCl (pH 7.5), 50 mM NaCl, 2.5 mM MgCl_2_, BSA (0.5 mg/ml), 40 μM biotin, and 1 mM DTT. When indicated, ADP, ATPγS, and ATP were added to both protein and buffer channels at a final concentration of 1 mM. Salt concentration was modified from 50 mM to 125, 300, or 500 mM NaCl in the buffer channel as specified in the text and figures. FE curves were performed at a speed of 1 μm/s. Compaction experiments were carried out at a constant force of 1 pN. For friction experiments, beads were moved 6 μm, back and forth, at a speed of 0.2 μm/s. SYTOX Orange (Invitrogen, Thermo Fisher Scientific) was used at a final concentration of 50 mM for DNA imaging, using a 532-nm wavelength laser. Force data were processed using Igor Pro 7 software (WaveMetrics), and images were processed using Adobe Photoshop CC.

### Western blot

For Western blot, 2 μg of purified complexes was run on NuPAGE 4 to 12% bis-tris gels (Thermo Fisher Scientific), transferred to Immobilon-P membranes (Millipore), and probed with anti-Strep (ab180957, Abcam, 1:5000) and anti-HA (3F10, Roche, 1:5000) antibodies in 5% milk–phosphate-buffered saline (PBS)/0.01% Tween overnight at 4°C. Membranes were then washed and incubated with horseradish peroxidase anti-rabbit (Santa Cruz Biotechnology, 1:40,000) and anti-rat (Jackson ImmunoResearch, 1:10,000) secondary antibodies, respectively, for 1 hour at room temperature. Immunoblots were developed using the Luminata Forte detection reagent (Millipore) and Hyperfilms ECL (GE Healthcare).

### Liquid chromatography–tandem mass spectrometry

Samples were processed by in-Stage Tip digestion (PreOmics GmbH, Planegg/Martinsried) following the manufacturer’s recommendation. Protein digests were solubilized in 30 μl of reconstitution buffer and transferred to autosampler vials for liquid chromatography–mass spectrometry analysis. Peptides were separated using an Ultimate 3000 RSLC nano–liquid chromatography system (Thermo Fisher Scientific) coupled to an LTQ Orbitrap Velos mass spectrometer (Thermo Fisher Scientific) via an EASY-Spray source. Sample volumes were loaded onto a trap column (Acclaim PepMap 100 C18, 100 μm × 2 cm) at 8 μl/min in 2% acetonitrile and 0.1% trifluoroacetic acid. Peptides were eluted online to an analytical column (EASY-Spray PepMap C18, 75 μm × 50 cm). Peptides were separated using a ramped 120-min gradient from 1 to 42% buffer B [buffer A: 5% dimethyl sulfoxide (DMSO), 0.1% formic acid; buffer B: 75% acetonitrile, 0.1% formic acid, 5% DMSO]. Eluted peptides were analyzed operating in positive polarity using a data-dependent acquisition mode. Ions for fragmentation were determined from an initial MS1 survey scan at 30,000 resolution [at mass/charge ratio (*m*/*z*) of 200] in the Orbitrap followed by CID (collision-induced dissociation) of the top 10 most abundant ions in the Ion Trap. MS1 and MS2 scan AGC targets were set to 1 × 10^6^ and 1 × 10^5^ for a maximum injection time of 50 and 110 ms, respectively. A survey scan *m*/*z* range of 350 to 1500 *m*/*z* was used, with CID parameters of isolation width 1.0 *m*/*z*, normalized collision energy of 35%, activation Q of 0.25, and activation time of 10 ms.

Data were processed using the MaxQuant software platform (v1.6.2.3) with database searches carried out by the in-built Andromeda search engine against the UniProt *S. cerevisiae* database (6729 entries, v.20180305). A reverse decoy database was created, and results were displayed at a 1% false discovery rate for peptide spectrum matches and protein identification. Search parameters included the following: trypsin, two missed cleavages, fixed modification of cysteine carbamidomethylation and variable modifications of methionine oxidation, asparagine deamidation, and protein N-terminal acetylation. Label-free quantification (LFQ) was enabled with an LFQ minimum ratio count of 2. “Match between runs” function was used with match and alignment time limits of 0.7 and 20 min, respectively. Protein and peptide identification and relative quantification outputs from MaxQuant were further processed in Microsoft Excel, with hits to the “reverse database,” “potential contaminants” (peptide list only), and “only identified by site” fields removed.

## Supplementary Material

http://advances.sciencemag.org/cgi/content/full/5/11/eaay6804/DC1

Download PDF

Table S1

Table S2

Movie S1

Movie S2

Movie S3

Movie S4

Movie S5

Movie S6

A conserved ATP- and Scc2/4-dependent activity for cohesin in tethering DNA molecules

## SUPPLEMENTARY MATERIALS

Supplementary material for this article is available at http://advances.sciencemag.org/cgi/content/full/5/11/eaay6804/DC1 Fig. S1. Topological loading of yeast cohesin on plasmid DNA. Fig. S2. Analysis of yeast cohesin on DNA curtains. Fig. S3. Intramolecular cohesin bridging requires ATP. Fig. S4. Purification of budding yeast cohesin ATPase mutant. Fig. S5. Permanent cohesin bridges are not displaced by physical stretching of λ-DNA. Fig. S6. Cohesin does not capture two λ-DNAs in sequential steps. Fig. S7. DNA friction experiments confirm the presence of cohesin complexes on extended λ-DNA. Fig. S8. Generation of permanent cohesin bridges using a quadrupole-trap optical tweezer. Fig. S9. Purification of human cohesin and yeast condensin. Fig. S10. Budding yeast condensin, but not cohesin, compacts λ-DNA against 1 pN stretching force. Table S1. Mass spectrometry analysis of cohesin wild type and ATPase mutant (Smc3-K38I) tetramer complexes and the loader complex Scc2-Scc4. Table S2. Mass spectrometry analysis of cohesin ATPase mutant (Smc3-K38I) tetramer peptides showing peptides containing the K38I mutation for SMC3. Movies S1 to S3. Time-lapse videos showing cohesin tethering. Movies S4 and S5. Time-lapse videos showing sliding of intermolecular bridges in a quadruple-trap optical tweezer. Movie S6. Time-lapse video showing pulling on intermolecular bridges in a quadruple-trap optical tweezer.

## Appendix

View/request a protocol for this paper from *Bio-protocol*.
