# Supplementary material for: A conserved ATP- and Scc2/4-dependent activity for cohesin in tethering DNA molecules
Source: Sci Adv. 2019 Nov 27;5(11):eaay6804. doi: 10.1126/sciadv.aay6804 (PMC6881171; doi:10.1126/sciadv.aay6804)
Supplement: http://advances.sciencemag.org/cgi/content/full/5/11/eaay6804/DC1 [file supp_5_11_eaay6804__index.html]

Science Advances | Science AdvancesAAASSearchScience AdvancesMenu

## Supplementary Materials

**The PDFset includes:**

- Fig. S1. Topological loading of yeast cohesin on plasmid DNA.
- Fig. S2. Analysis of yeast cohesin on DNA curtains.
- Fig. S3. Intramolecular cohesin bridging requires ATP.
- Fig. S4. Purification of budding yeast cohesin ATPase mutant.
- Fig. S5. Permanent cohesin bridges are not displaced by physical stretching of λ-DNA.
- Fig. S6. Cohesin does not capture two λ-DNAs in sequential steps.
- Fig. S7. DNA friction experiments confirm the presence of cohesin complexes on extended λ-DNA.
- Fig. S8. Generation of permanent cohesin bridges using a quadrupole-trap optical tweezer.
- Fig. S9. Purification of human cohesin and yeast condensin.
- Fig. S10. Budding yeast condensin, but not cohesin, compacts λ-DNA against 1 pN stretching force.
- Legends for tables S1 and S2
- Legends for movies S1 to S6

Download PDF

**Other Supplementary Material for this manuscript includes the following:**

- Table S1 (Microsoft Excel format). Mass spectrometry analysis of cohesin wild type and ATPase mutant (Smc3-K38I) tetramer complexes and the loader complex Scc2-Scc4.
- Table S2 (Microsoft Excel format). Mass spectrometry analysis of cohesin ATPase mutant (Smc3-K38I) tetramer peptides showing peptides containing the K38I mutation for SMC3.
- Movie S1 (.mp4 format). Time-lapse videos showing cohesin tethering.
- Movie S2 (.mp4 format). Time-lapse videos showing cohesin tethering.
- Movie S3 (.mp4 format). Time-lapse videos showing cohesin tethering.
- Movie S4 (.mp4 format). Time-lapse videos showing sliding of intermolecular bridges in a quadruple-trap optical tweezer.
- Movie S5 (.mp4 format). Time-lapse videos showing sliding of intermolecular bridges in a quadruple-trap optical tweezer.
- Movie S6 (.mp4 format). Time-lapse video showing pulling on intermolecular bridges in a quadruple-trap optical tweezer.

**Files in this Data Supplement:**

- Adobe PDF - aay6804\_SM.pdf
