# Supplementary material for: A conserved ATP- and Scc2/4-dependent activity for cohesin in tethering DNA molecules
Source: Sci Adv. 2019 Nov 27;5(11):eaay6804. doi: 10.1126/sciadv.aay6804 (PMC6881171; doi:10.1126/sciadv.aay6804)
Supplement: Download PDF [file aay6804_SM.pdf]

Science Advances
AAAS

advances.sciencemag.org/cgi/content/full/5/11/eaay6804/DC1

Supplementary Materials for

A conserved ATP- and Scc2/4-dependent activity for cohesin in tethering DNA molecules

Pilar Gutierrez-Escribano, Matthew D. Newton, Aida Llauró, Jonas Huber, Loredana Tanasie, Joseph Davy, Isabel Aly, Ricardo Aramayo, Alex Montoya, Holger Kramer, Johannes Stigler*, David S. Rueda*, Luis Aragon*

*Corresponding author Email: stigler@genzentrum.lmu.de (J.S.); david.rueda@imperial.ac.uk (D.S.R.); luis.aragon@csc.mrc.ac.uk (L.A.)

Published 27 November 2019, *Sci. Adv.* **5**, eaay6804 (2019)
DOI: 10.1126/sciadv.aay6804

The PDF file includes:

Fig. S1. Topological loading of yeast cohesin on plasmid DNA.
Fig. S2. Analysis of yeast cohesin on DNA curtains.
Fig. S3. Intramolecular cohesin bridging requires ATP.
Fig. S4. Purification of budding yeast cohesin ATPase mutant.
Fig. S5. Permanent cohesin bridges are not displaced by physical stretching of λ-DNA.
Fig. S6. Cohesin does not capture two λ-DNAs in sequential steps.
Fig. S7. DNA friction experiments confirm the presence of cohesin complexes on extended λ-DNA.
Fig. S8. Generation of permanent cohesin bridges using a quadrupole-trap optical tweezer.
Fig. S9. Purification of human cohesin and yeast condensin.
Fig. S10. Budding yeast condensin, but not cohesin, compacts λ-DNA against 1 pN stretching force.
Legends for tables S1 and S2
Legends for movies S1 to S6

Other Supplementary Material for this manuscript includes the following:

(available at advances.sciencemag.org/cgi/content/full/5/11/eaay6804/DC1)

Table S1 (Microsoft Excel format). Mass spectrometry analysis of cohesin wild type and ATPase mutant (Smc3-K38I) tetramer complexes and the loader complex Scc2-Scc4.
Table S2 (Microsoft Excel format). Mass spectrometry analysis of cohesin ATPase mutant (Smc3-K38I) tetramer peptides showing peptides containing the K38I mutation for SMC3.
Movie S1 (.mp4 format). Time-lapse videos showing cohesin tethering.
Movie S2 (.mp4 format). Time-lapse videos showing cohesin tethering.

Movie S3 (.mp4 format). Time-lapse videos showing cohesin tethering.
Movie S4 (.mp4 format). Time-lapse videos showing sliding of intermolecular bridges in a quadruple-trap optical tweezer.
Movie S5 (.mp4 format). Time-lapse videos showing sliding of intermolecular bridges in a quadruple-trap optical tweezer.
Movie S6 (.mp4 format). Time-lapse video showing pulling on intermolecular bridges in a quadruple-trap optical tweezer.

Supplementary Figure 1

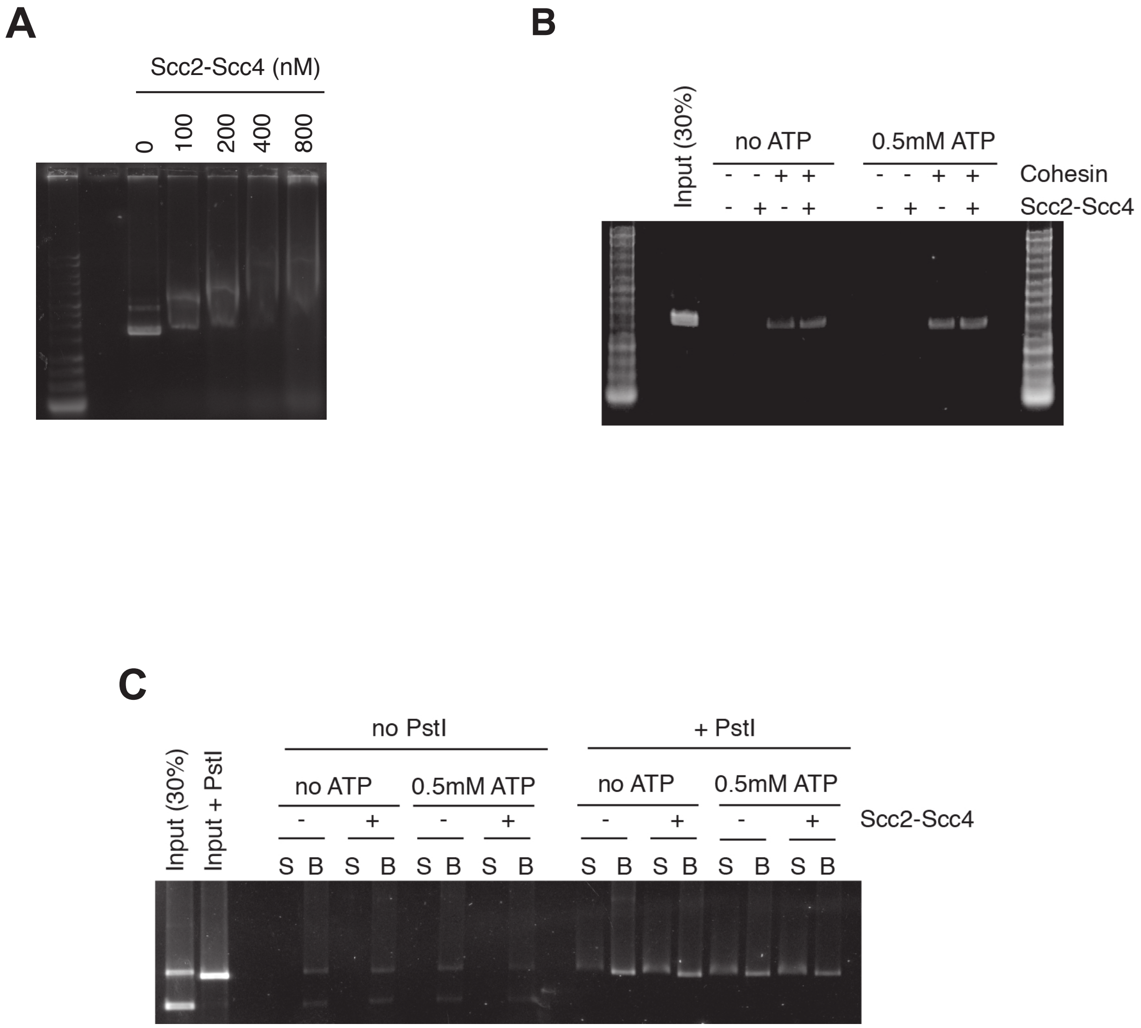

Fig. S1. Topological loading of yeast cohesin on plasmid DNA. A. Electrophoretic mobility shift assays using pUC19 as substrate and the indicated Scc2-Scc4 complex concentrations, showing that purified Scc2/Scc4 complex has DNA binding activity. **B.** Agarose gel electrophoresis showing recovered DNA after cohesin loading and immunoprecipitation with and without 0.5mM ATP both in the presence and absence of Scc2/4 complex. Topological assays were done as in (6). **C**. Gel image of recovered DNA in supernatant (S) and cohesin-bound bead (B) fractions after linearisation of immunoprecipitated cohesin-bound DNA by PstI digestion.

Supplementary Figure 2

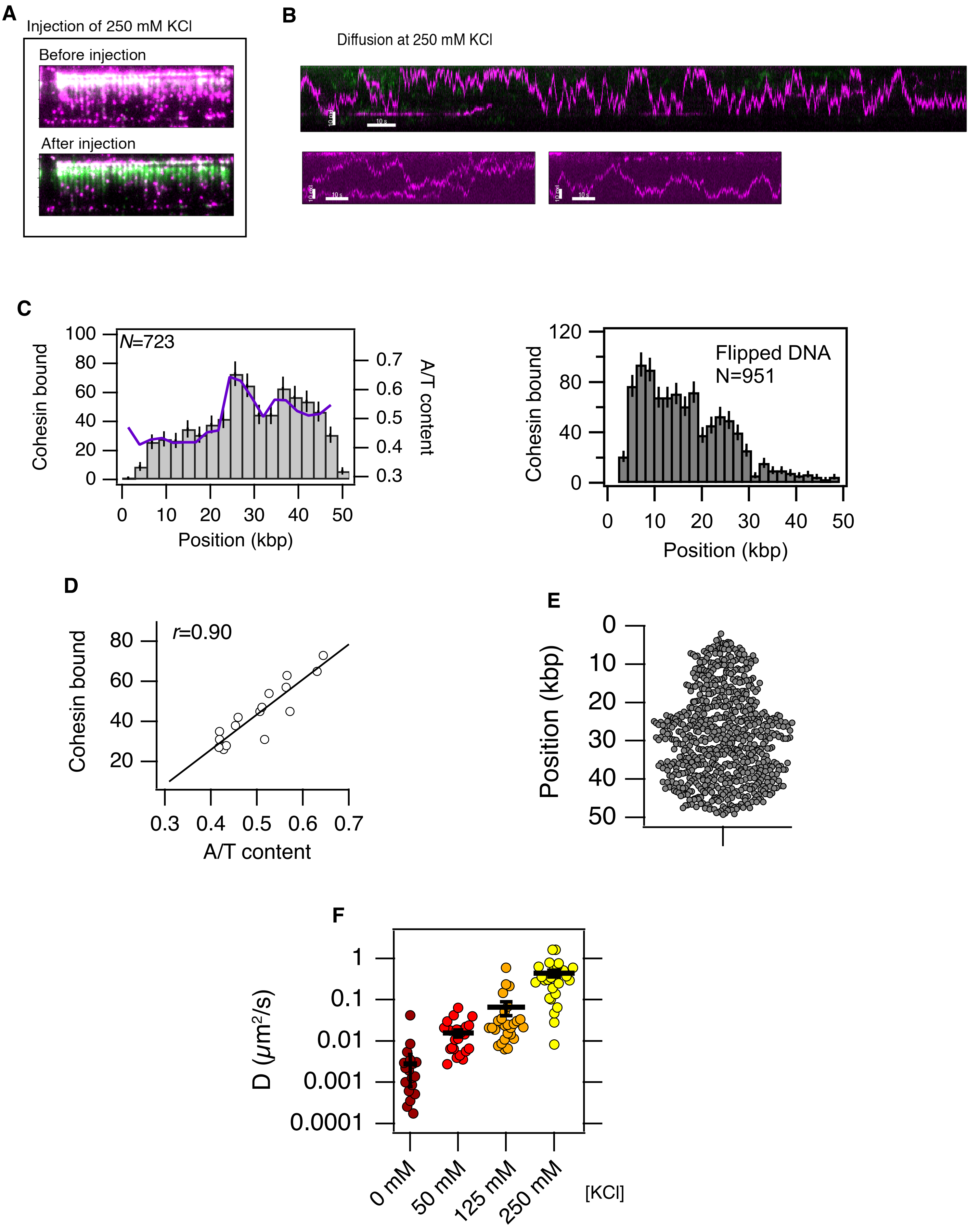

Fig. S2. Analysis of yeast cohesin on DNA curtains. A. Image of cohesin tagged with quantum dots (QD) (magenta) bound at 25 mM KCl to λ-DNA before injection of high ionic strength buffer (top). Image of cohesin (magenta) bound to λ-DNA (green) after injection of 250 mM KCl buffer (bottom). **A**. Representative kymograph illustrating cohesin diffusion at 250 mM KCl. **C**. Distribution of bound cohesins on λ-DNA. A/T content is indicated. Error bars: 68% confidence intervals. Distribution of bound cohesins on flipped λ-DNA is shown (right graph). **D**.Correlation between cohesin localisation and A/T nucleotide content on λ-DNA (Pearson's r = 0.90). **E**. Initial binding positions of individual cohesin to λ-DNA. **F**. Diffusion coefficients for cohesin movement in buffers of different of ionic concentration.

Supplementary Figure 3

A

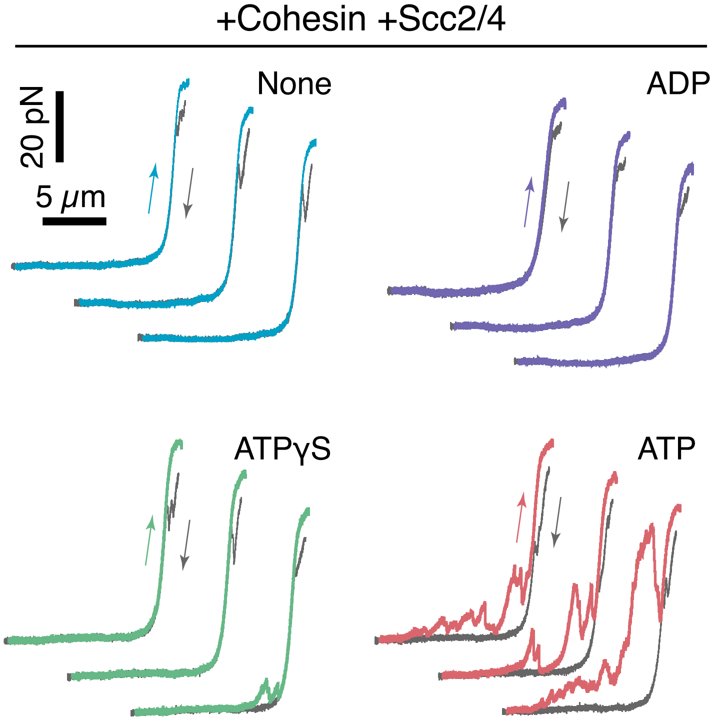

B

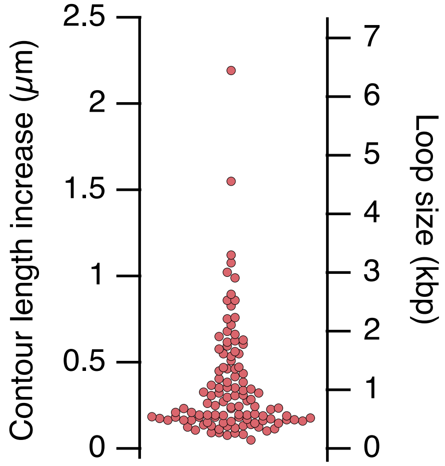

Fig. S3. Intramolecular cohesin bridging requires ATP. A. FE curves for λ-DNA pre-incubated with 1 nM cohesin, 2.5 nM Scc2-Scc4 complex and ATP analogues. After capturing a single DNA molecule between two optically trapped beads, DNA was incubated in the presence of protein in a relaxed conformation (3 μm bead distance) for 30s in 50mM NaCl and then moved to a buffer channel with 50mM NaCl for extension and measurements. Only ATP exhibits DNA bridging rupture events. Note that one of the FE curves (first curve in the ATP set) was used to illustrate bond-rupture events in Fig. 2A. **B**. Distribution of cohesin-induced DNA bridge loop sizes calculated from bond-rupture events in FE curves.

Supplementary Figure 4

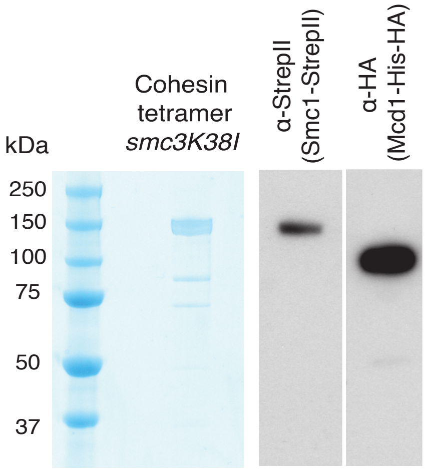

Fig. S4. Purification of budding yeast Cohesin ATPase mutant. Purified cohesin tetramer containing Smc1, Smc3-K38I, Mcd1 and Scc3 was analysed by SDS-PAGE electrophoresis followed by Coomassie Blue staining. Western analysis showing Smc1-strep and Mcd1 HA is included.

Supplementary Figure 5

300 mM NaCl

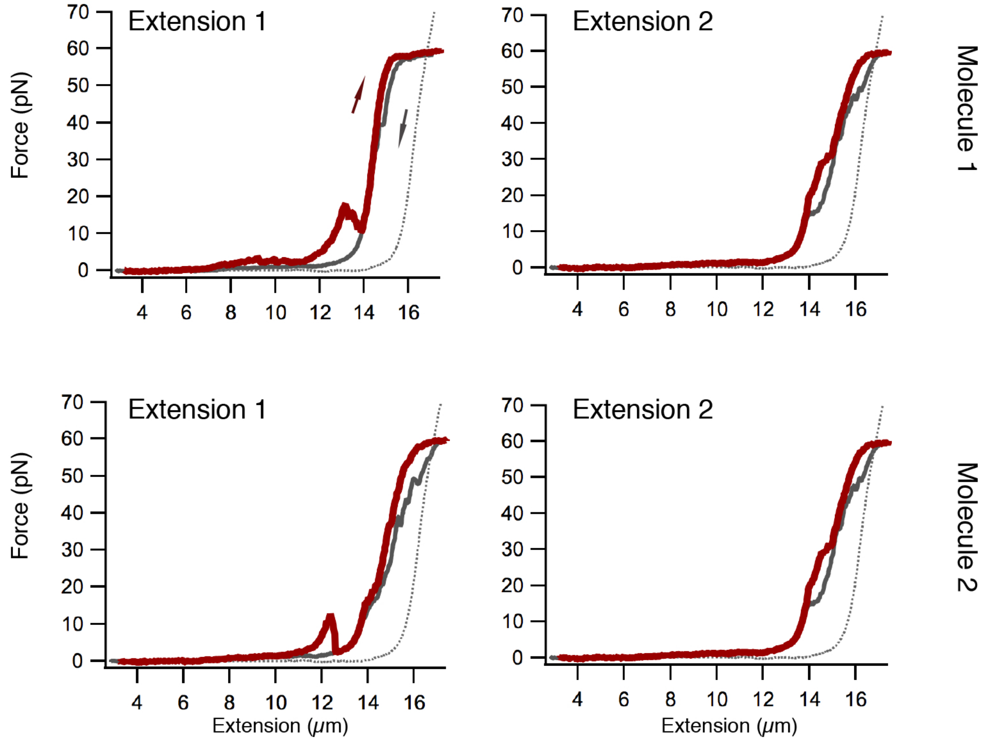

500 mM NaCl

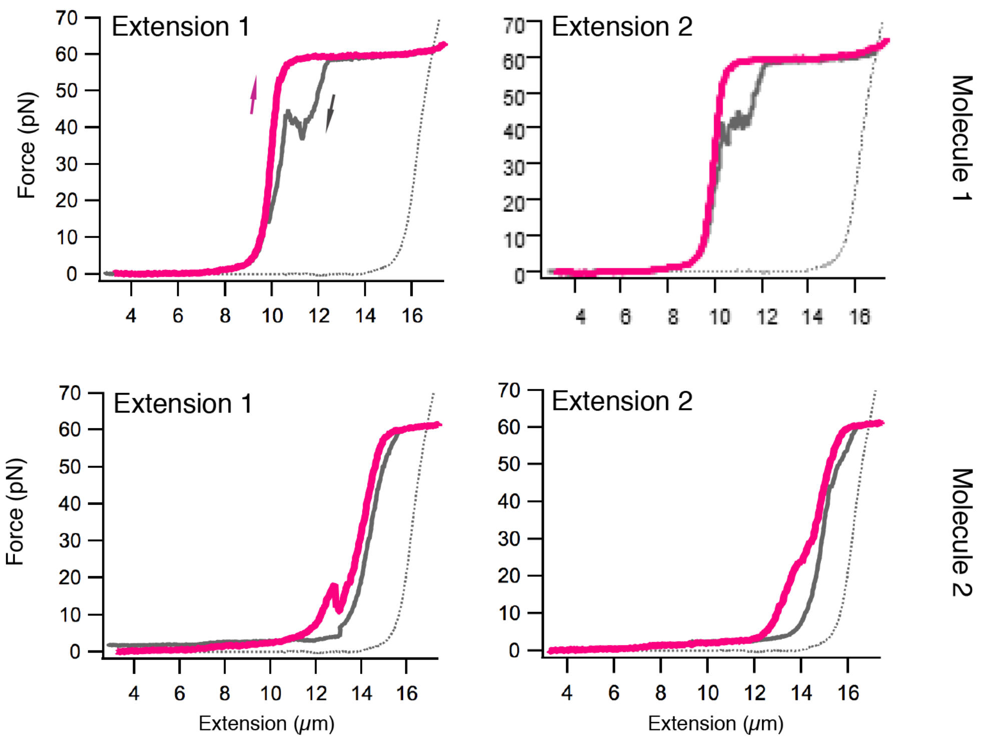

Fig. S5. Permanent cohesin bridges are not displaced by physical stretching of λ-DNA.

FE curves during sequential extension and relaxation cycles at 300mM and 500mM NaCl.

After capture of λ-DNA between the two optically trapped beads, DNA is relaxed (3μm bead distance) and incubated for 30 seconds in the protein channel (1 nM cohesin and 2.5 nM complex and 1 mM ATP in 50mM NaCl). DNA is moved to a buffer channel (either 300mM NaCl or 500mM NaCl as indicated) before re-extension to test for DNA bridges. After confirmation of the bridges and full extension of the molecule (FE curves on the left), the DNAs are relaxed in the same channel (either 300mM NaCl or 500mM NaCl as indicated) and re-extended for a second time. FE curves of the second re-extension (FE curves on the right) show that the DNA bridge has not been displaced. Two independent molecules are shown. The first extension is also shown in Fig. 3D-F.

Supplementary Figure 6

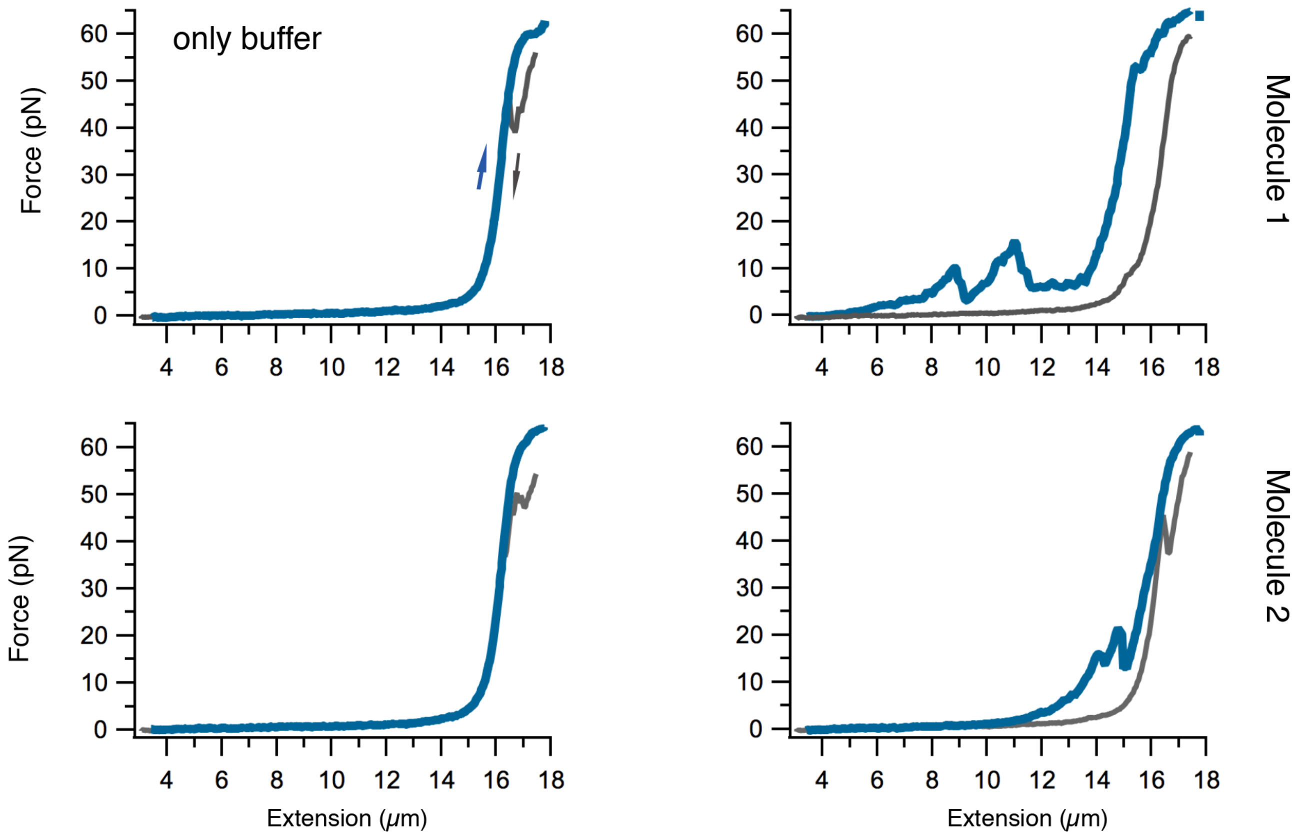

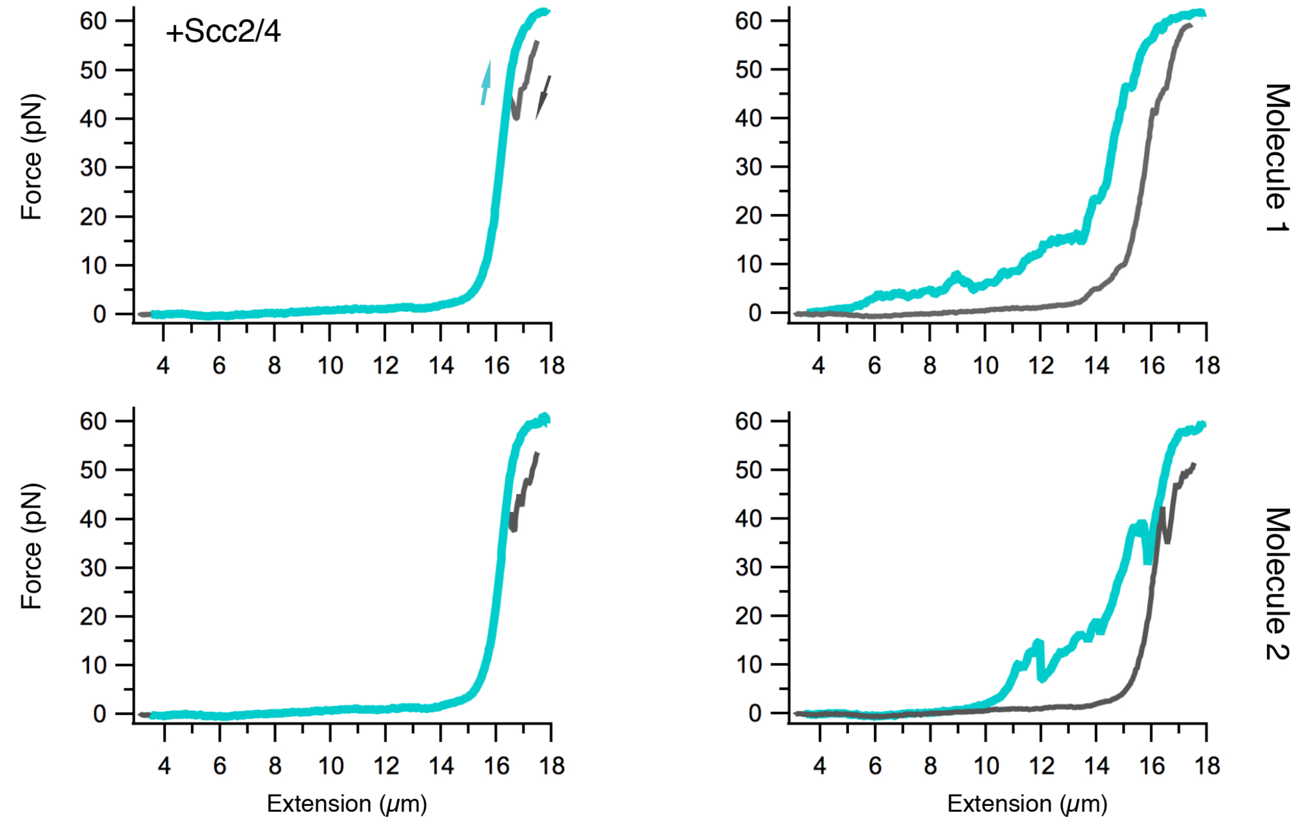

Fig. S6. Cohesin does not capture two λ-DNAs in sequential steps. λ-DNA incubated with 1nM cohesin, 2.5 nM Scc2-Scc4 complex and 1mM ATP in an extended conformation. The DNAs were moved to a buffer channel (50mM NaCl) in the presence of 1mM ATP (buffer only - dark blue- top two left FE curves) or 2.5nM Scc2-Scc4 complex and 1mM ATP (+Scc2/4 - light blue – bottom two left FE curves). The λ-DNA molecules were then incubated in a relaxed position (3μm bead distance) for 30s. DNAs were then moved to an only buffer channel (125mM NaCl containing 1mM ATP) and re-extended (right FE curves; top - only buffer - during the first relaxation, bottom buffer plus Scc2/4 during the first relaxation). The same molecules were then relaxed (3μm bead distance) and incubated (for 30s) with 1nM cohesin, 2.5 nM Scc2-Scc4 complex and 1mM ATP in 50mM NaCl. Finally, the DNA was moved to a different channel with 1mM ATP in 125mM NaCl and re-extended. FE curves of the final re-extension are shown (left FE curves; top- only buffer - during the first relaxation, -bottom- buffer plus Scc2/4 during the first relaxation). Only FE curves incubated with cohesin in a relaxed conformation show the presence of DNA bridging rupture events (i.e. FE curves on the right).

Supplementary Figure 7

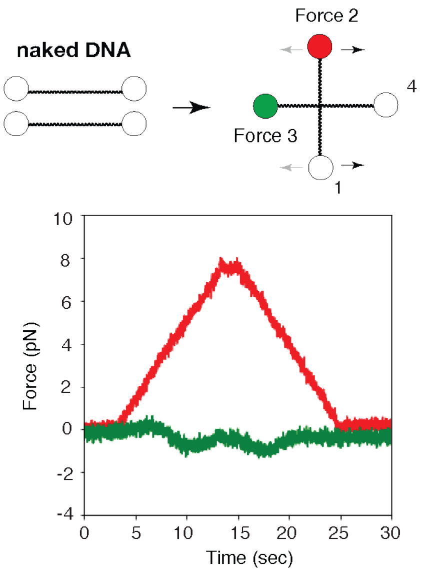

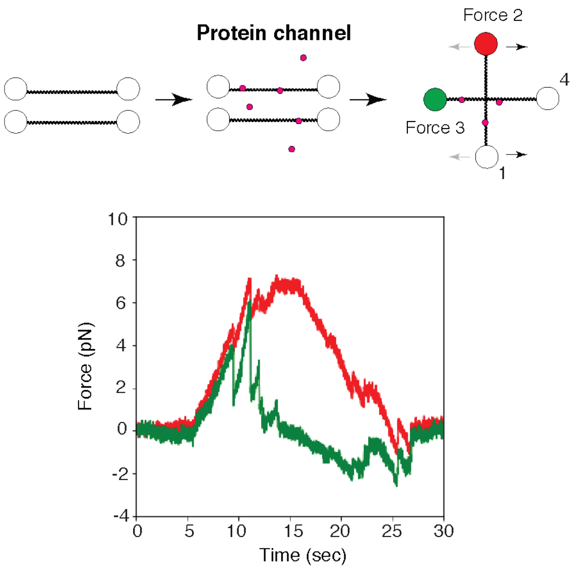

Fig. S7. DNA friction experiments confirm the presence of cohesin complexes on extended λ-DNA. Friction experiments were performed on a quadruple-trap optical tweezer system as described in (*11*). Two molecules of λ-DNA were tethered independently between two pairs of beads. Beads were then moved to the protein channel, containing 1nM cohesin and 2.5nM Scc2/4 complex in 50mM NaCl plus 1mM ATP, and incubated separated in an extended position (~14 μm bead distance). After incubation, the beads were moved to the buffer channel containing 125mM NaCl and 1mM ATP and crossed, so contact between both DNA molecules was established. Beads 1 and 2 were then moved simultaneously in the x axis to cause the sliding of one of the DNA molecules on top of the other. Forces in both beads 2 (sliding DNA molecule) and 3 (static DNA molecule) were recorded. A negative control with naked DNA is included (left panel). The presence of bound protein was identified by the appearance of abrupt changes in both force 2 and force 3 caused by the movement of beads 1 and 2 (right panel). For graphical representation, force data were downsampled to 100Hz.

Supplementary Figure 8

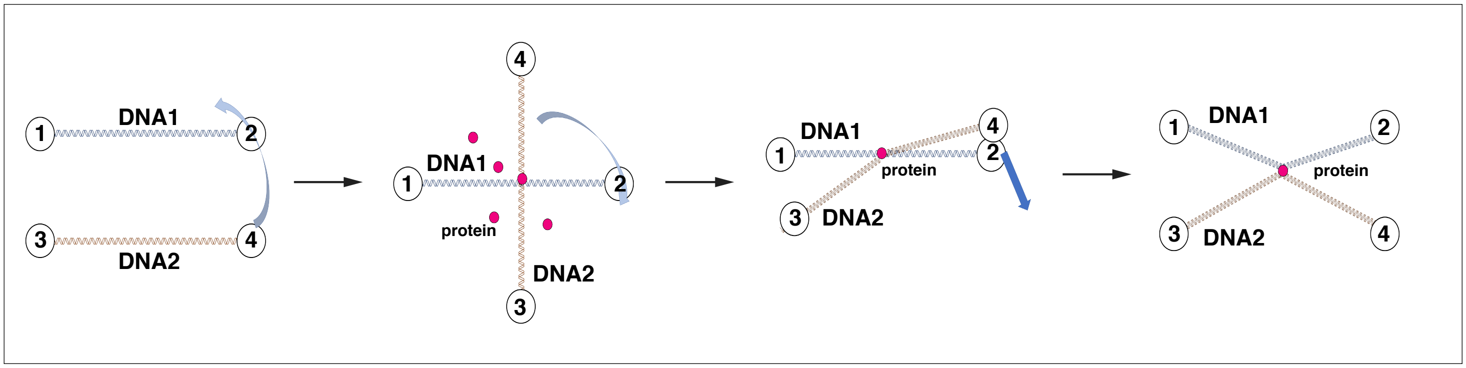

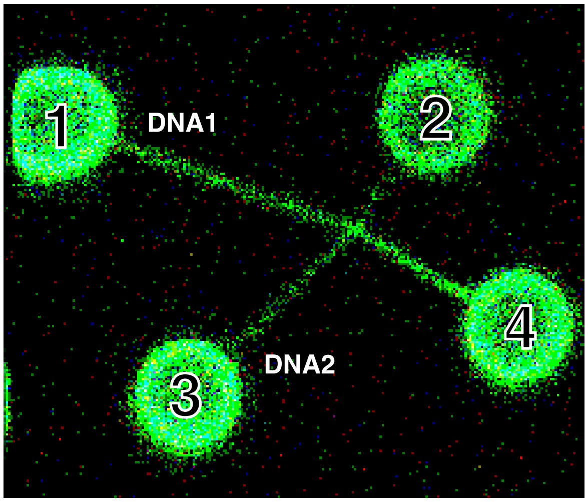

Fig. S8. Generation of permanent cohesin bridges using a quadrupole-trap optical tweezer.
A. Schematic representation of the experimental design for the quadrupole trap optical tweezer to generate permanent cohesin bridges. First, a pair of λ-DNAs (DNA1 and DNA2) are trapped between two pairs of beads (beads 1 and 2 trap DNA1, while beads 3 and 4 trap DNA2) and kept extended (15μm bead distance). DNA2 is manipulated using beads 3 and 4, moved over DNA1 and positioned at a 90 angle, then relaxed (3μm bead distance). The crossed DNAs are moved to a channel containing 1 nM cohesin and 2.5 nM Scc2-Scc4 complex and 1mM ATP in 50mM NaCl and incubated for 30s. The crossed DNAs are moved to a channel containing 1mM ATP in 300mM NaCl and DNA2 is extended and moved back to its original position using beads 3 and 4. We used 50nM of SYTOX Orange to visualise the bridged DNA (right image). DNA molecules are in green, beads are numbered.

Supplementary Figure 9

A

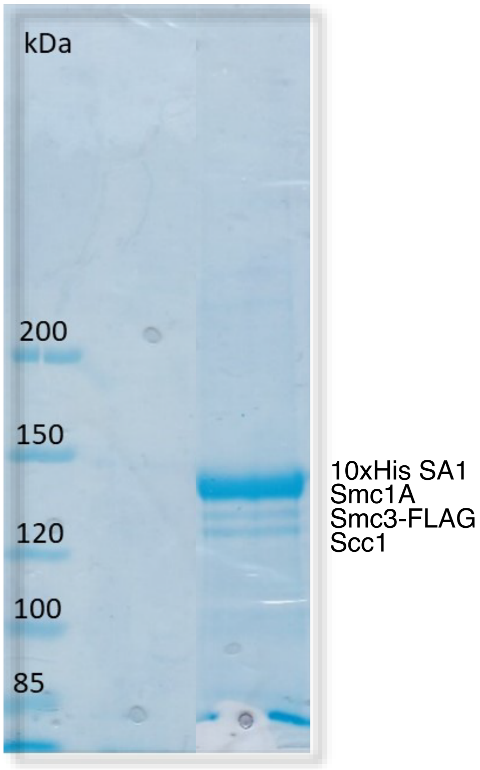

B

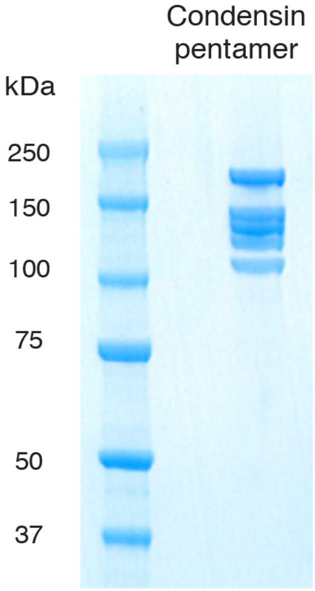

C

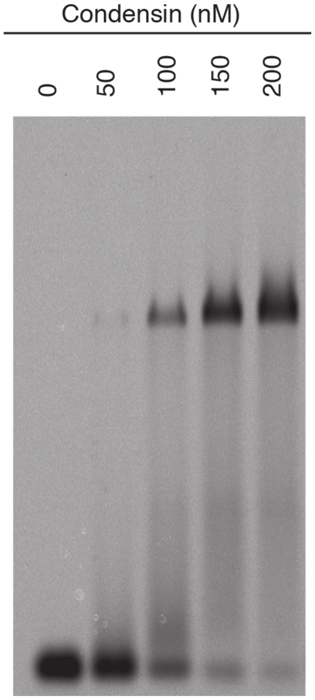

Fig. S9. Purification of human Cohesin and yeast condensin. A. Purified cohesin tetramer containing hSmc1A, Smc3-FLAG, Scc1 and 10xHis SA1 was analysed by SDS-PAGE electrophoresis followed by Coomassie Blue staining. Purification was done using biGBac vector (pBIG1c) as described previously (*3*). **B.** Purified condensin pentamers containing Smc2, Smc4, Brn1 and Ycs4 and Ycg1 was analysed by SDS-PAGE electrophoresis followed by Coomassie Blue staining. Purifications were done as in (*1, 2*). **C.** Electrophoretic mobility shift assays with a 6-carboxyfluorescein–labelled 41-bp dsDNA substrate (100nM) and the indicated protein concentrations.

Supplementary Figure 10

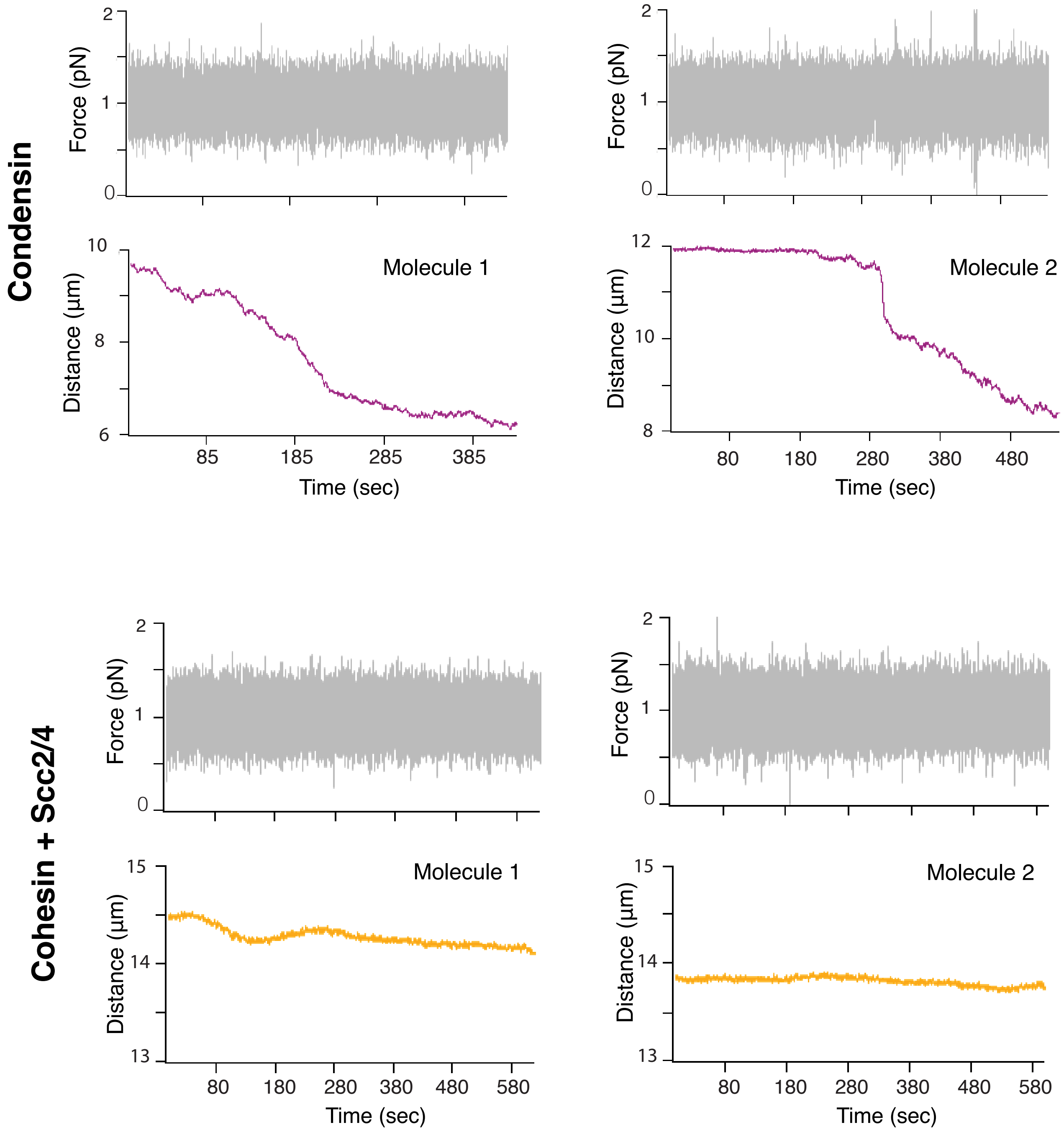

Fig. S10. Budding yeast condensin, but not cohesin, compacts λ-DNA against 1 pN stretching force. Examples of DNA compaction traces for λ-DNA molecule extended using a force of 1pN (top). The DNAs were tethered between two beads. One bead was clamped (fixed) while a 5pN force was applied to the second bead to maintain the molecule extended. The DNA was then incubated in the presence of 1nM condensin (1mM ATP in 50mM NaCl) (top- condensin - magenta traces) or 1nM Cohesin and 2.5 nM Scc2-Scc4 complex (1mM ATP in 50mM NaCl) (bottom -cohesin -- yellow traces). Extended DNAs were then moved to a different channel containing 1mM ATP in 50mM NaCl and the extension force was reduced to 1pN. The distance between the beads was recorded over time. Only condensin was able to reduce the distance between the beads over time consistent with a DNA compaction activity. Two independent molecule traces for each complex is shown. An additional trace is shown in Fig. 4F-H. For graphical representation, force data were downsampled to 100Hz.

Table S1. Mass spectrometry analysis of cohesin wild type and ATPase mutant (Smc3-K38I) tetramer complexes and the loader complex Scc2-Scc4.

Protein identifications for cohesin tetramer (Wt and K38I) and Scc2-Scc4 purifications.

Table S2. Mass spectrometry analysis of cohesin ATPase mutant (Smc3-K38I) tetramer peptides showing peptides containing the K38I mutation for SMC3.

Identifications of K38I peptides in purifications of cohesin ATPase mutant (Smc3-K38I).

Movies S1 to S3. Time-lapse videos showing cohesin tethering.

Time lapse videos showing cohesin tethering. Individual pairs of double-tethered DNA curtains (DNA molecules are in green) bound by cohesin is in magenta are shown.

Movies S4 and S5. Time-lapse videos showing sliding of intermolecular bridges in a quadruple-trap optical tweezer.

Time lapse videos showing sliding of intermolecular bridges in a quadruple-trap optical tweezer. Images are shown in Fig. 4C. DNA molecules are in green.

Movie S6. Time-lapse video showing pulling on intermolecular bridges in a quadruple-trap optical tweezer.

Time lapse video showing pulling on intermolecular bridges in a quadruple-trap optical tweezer. Images are shown in Fig. 4D. DNA molecules are in green.
